# Innovations and Patent Trends in the Development of USFDA Approved Protein Kinase Inhibitors in the Last Two Decades

**DOI:** 10.3390/ph14080710

**Published:** 2021-07-22

**Authors:** Mohd. Imran, Syed Mohammed Basheeruddin Asdaq, Shah Alam Khan, Dhanalekshmi Unnikrishnan Meenakshi, Abdulhakeem S. Alamri, Walaa F. Alsanie, Majid Alhomrani, Yahya Mohzari, Ahmed Alrashed, Mohammed AlMotairi, Eman H. Alkhaldi, Abeer K. Alorabi, Ahmed Subeh Alshrari, Mohammad Tauseef, Abida, Saleh I. Alaqel, Ozair Alam, Md. Afroz Bakht

**Affiliations:** 1 Department of Pharmaceutical Chemistry, Faculty of Pharmacy, Northern Border University, Arar 91911, Saudi Arabia; aqua_abkhan@yahoo.com (A.); saleh_alagel@hotmail.com (S.I.A.); 2 Department of Pharmacy Practice, College of Pharmacy, AlMaarefa University, Dariyah, Riyadh 13713, Saudi Arabia; 3 College of Pharmacy, National University of Science and Technology, Muscat 130, Oman; shahalam@nu.edu.om (S.A.K.); dhanalekshmi@nu.edu.om (D.U.M.); 4 Department of Clinical Laboratory Sciences, The Faculty of Applied Medical Sciences, Taif University, Taif 21944, Saudi Arabia; a.alamri@tu.edu.sa (A.S.A.); w.alsanie@tu.edu.sa (W.F.A.); m.alhomrani@tu.edu.sa (M.A.); 5 Centre of Biomedical Sciences Research (CBSR), Deanship of Scientific Research, Taif University, Taif 21944, Saudi Arabia; 6 Clinical Pharmacy Department, King Saud Medical City, Riyadh 12746, Saudi Arabia; Yali2016@hotmail.com; 7 Pharmaceutical Services Administration, Inpatient Department, Main Hospital, King Fahad Medical City, Riyadh 11564, Saudi Arabia; emadasdaq@gmail.com; 8 Department of Clinical Pharmacy, King Fahad Medical City, Riyadh 11564, Saudi Arabia; mhospital1920@gmail.com; 9 Pharmaceutical Care Services, King Saud Medical City, Riyadh 12746, Saudi Arabia; emanalka62@gmail.com; 10 Pharmaceutical Care Services, King Salman Specialist Hospital, Hail 55471, Saudi Arabia; Akalorabi@moh.gov.sa; 11 Department of Biological Sciences, Faculty of Science, Northern Border University, Arar 91431, Saudi Arabia; alshrari@live.com; 12 Department of Pharmaceutical Sciences, College of Pharmacy, Chicago States University, Chicago, IL 60607, USA; mtauseef@csu.edu; 13 Medicinal Chemistry and Molecular Modelling Lab., Department of Pharmaceutical Chemistry, School of Pharmaceutical Education and Research, Jamia Hamdard, New Delhi 110062, India; dr.ozairalam@gmail.com; 14 Department of Chemistry, College of Science and Humanities, Prince Sattam Bin Abdulaziz University, Al-Kharj 11942, Saudi Arabia; bakhtpharm@gmail.com

**Keywords:** protein kinase inhibitors, USFDA, cancer, inflammation, patent review, generic product

## Abstract

Protein kinase inhibitors (PKIs) are important therapeutic agents. As of 31 May 2021, the United States Food and Drug Administration (USFDA) has approved 70 PKIs. Most of the PKIs are employed to treat cancer and inflammatory diseases. Imatinib was the first PKI approved by USFDA in 2001. This review summarizes the compound patents and the essential polymorph patents of the PKIs approved by the USFDA from 2001 to 31 May 2021. The dates on the generic drug availability of the PKIs in the USA market have also been forecasted. It is expected that 19 and 48 PKIs will be genericized by 2025 and 2030, respectively, due to their compound patent expiry. This may reduce the financial toxicity associated with the existing PKIs. There are nearly 535 reported PKs. However, the USFDA approved PKIs target only about 10–15% of the total said PKs. As a result, there are still a large number of unexplored PKs. As the field advances during the next 20 years, one can anticipate that PKIs with many scaffolds, chemotypes, and pharmacophores will be developed.

## 1. Introduction

Protein kinases (PKs) are ubiquitous intracellular and cell surface enzymatic proteins that selectively catalyzes phosphate group’s relocation from ATP, GTP, and other phosphate donors to protein substrates [1]. The PKs mainly catalyze the relocation of a γ-phosphatase group of ATP to the oxygen atom of the -OH group of threonine, serine, and tyrosine residues in peptides/polypeptides, thereby making a conformational variation from an inactive to an active form [1,2]. They constitute an extensive family of structurally related enzymes that are known to be implicated in almost all the signal transduction activities, frequently with cascades of phosphorylation proceedings taking place within the cell [3]. The signal transduction involves the reversible phosphorylation of proteins that helps to regulate mature proteins by altering their structure and function [4,5]. To date, nearly 535 human PKs have been identified [6], wherein more than 478 belong to a superfamily whose catalytic domains are sequentially interrelated. These PKs are additionally categorized into groups, families, and subfamilies established on their biochemical activities. The main two classifications are Serine/threonine PKs and Tyrosine-specific PKs [5]. The seven significant groups with the description of families, subfamilies, and functions are listed in Table 1.

TKs form a distinct group, which phosphorylates proteins on tyrosine, whereas others phosphorylate serine and threonine residues. In addition to this category, there are atypical kinases, which are not related to any sequence resemblance to characteristic kinases but are well recognized for their enzymatic activity similar to specific kinases. Some kinases are believed to lack the catalytic domain for effective phosphorylation and are called pseudokinases. Still, they are distributed across all kinase families, indicating that an absence of catalysis is not a formal barricade to the evolution of unique or irreplaceable biological functions [7].

**Table 1:** Families and subfamilies of PKs.

S. No.	Kinase	Families	Subfamilies	Functions
Serine/Threonine-Specific Protein Kinases				
1	AGC	PKA, PKG, PKC, DMPK, NDR, AKT, SGK, RSK, PKN, GRK, PDK1, RSKR, RSKL, MAST	DMPK: GEK, ROCK, CRIK PKC: Alpha, Delta, Gamma, Epsilon RSK: MSK, P70 RSKL: RSKL1, RSKL2 MAST: MAST1, MAST 2, MAST3, MAST4, MASTL	They are implicated in various cellular activities and are prospective targets to treat cancer, inflammation, viral infections, obesity, diabetes, and neurological disorders [8]
2	CAMK	Calcium/calmodulin-dependent protein kinase-CAMK1, Unique VACAMKL, PSK, DAPK, MLCK, TRIO, CASK, CAMK2, PHK, DCAMKL, MAPKAPK, CAMKL, TSK, PIM, TRB1, Unique STK33, PKD, RAD53	MAPKAPK: MNK, MAPKAPK1, MAPKAPK2, MAPKAPK3, JNK CAMKL: AMPK, BRSK, MELK, MARK, QIK, NUAK, NIMI, SNRK, PASK, CHK1, LKB1, HUNK	They are implicated in the phosphorylation of transcription factors and the control of gene expression. They also control the life cycle of the cell [9]
3	CK1	Casein kinase 1, TTBK, VRK	-	They are involved in the phosphorylation of significant governing molecules in cellular translation/transcription, cell–cell adhesion, and receptor coupled signal transduction. They control main signaling trails, particularly in cancer evolution [10]
4	CMGC	CDK, MAPK, GSK3, CLK families, CDKL, CLK, RCK, DYRK	-	Critical role in cell cycle regulation and intracellular signal transduction [11]
5	STE	Homologs of yeast Sterile 7/MAP3K, Sterile 11/MAP2K, Sterile 20/MAP4K	MAP4K: FRAY, STLK, PAKA, PAKB, MST, YSK, TAO, MSN, NINAC, KHS, SLK	Crucial role in MAP kinase pathways, which require a sequential PK reaction to activate the next kinase in the pathway, especially in cascade process [12]
Tyrosine-Specific Protein Kinases				
6	TK	Tyrosine kinase	Receptor Tyrosine Kinases (RTKs): EGFR, EPH, SEV, ALK, TRK, INSR, CCK4, AXL, VEGFR, FGFR, MUSK, LMR, DDR, ROR, TIE, SEF, PDGFR, RET, MET, RYK	They play a vital role in controlling cellular differentiation, cell division, and morphogenesis. They primarily act as growth factor receptors and in downstream signaling [13]
			Non-Receptor Tyrosine Kinases (nRTKs): CSK, JAK, SRC (SFKs, BCR), BTK, ACK, SYK, FER, TEC, ABL, FAK	They are involved in signaling cascades, particularly those implicated in growth hormone and cytokine signaling. Some of them are involved in synaptic transmission, myelination, axon guidance, and oligodendrocyte formation [13]
7	TKL	Tyrosine kinase-like	IRAK, MLKL, LIMK, TESK, LRRK, ALK, ACTR, TGFR, MISR, BMPR, RAF, KSR, TAK, ILK, DLK, LZK, MLK, ZAK, RIPK, ANKRP, SGK, RIPK	They control apoptosis, cell differentiation/growth, angiogenesis, vascular development, and the protective response against pathogens [5,14]

PKs perform a significant function in signal transduction and control of most cellular processes, including cell growth, differentiation, proliferation, angiogenesis, apoptosis, cytoskeletal arrangement, regulation of metabolic reactions, membrane transport, and motility, etc. [6]. Non-catalytic functions of PKs are also essential and include the allosteric effect, subcellular targeting, the scaffolding of protein complexes, competition for protein interactions, and DNA binding [15]. Because PKs regulate most fundamental biological processes, any dysregulation, genetic alteration, and abrupt change in kinase function are typically linked with pathological conditions such as cancer, immunologic, neurological, cardiovascular, and metabolic disorders [3,5]. Hence, manipulation of PKs signaling pathway, regulation, and inhibition constitutes important clinical targets for pharmacological intervention and thus for the identification and development of Protein Kinase Inhibitors (PKIs) to manage and treat several chronic diseases [4,6,16]. Over the past two decades, approximately 1/5th-1/3rd drug discovery programs worldwide have targeted PKs for the drug development of various illnesses. 

Kinase mutation frequency is much less, and thus targeting kinases could be helpful in life-saving therapies especially for cancer. A well-known example is receptor tyrosine kinase ALK where gene fusion between EML4 and ALK occurs only in 5% of NSCLC patients and therefore many patients responded to the kinome therapy effectively. Identification of additional effective kinome targets will therefore represent an Achilles heel in a subset of cancer. The use of bioinformatics tools in predicting the likelihood that a given mutation will alter the function of a kinase will be essential in pinpointing cancer-associated kinases [17].

There are about 175 kinase drugs under clinical trials and newer targets are also under evaluation including AKT, Aurora kinases, CHEK1, and CDK1. However, most of the drugs under investigation are well known for targeting EGFR, VEGFR, PI3K, and mTOR [18]. Even though CAMK, CK1, or AGC kinases groups are well-known and evidenced as the primary targets for cancer, there are no investigational drugs that target these kinases are enrolled. So far only 8% of the entire kinome has been effectively “drugged” and a quarter of human kinases are vastly understudied [19]. A wide-ranging scoring system to rank and prioritize clinically relevant kinase targets of different solid tumor cancers from The Cancer Genome Atlas (TCGA) has been developed [19].

Successful applications and deep insights into the ever-diversifying therapeutic space occupied by kinase targets are also explored. For effective target validation and to avoid complicating off-target mediated response it is essential to achieve the desired selectivity while targeting kinases, though it is still an ongoing challenge. The application of large-scale omics data has been modernized to combine multiple parameters to evaluate the protein’s potential as a drug target or biomarker [19]. 

In recent years, intricately selective kinase chemical probes have been generated by the exploitation of unique pockets using molecular modeling and bioinformatics, prioritizing the ligand-efficient leads and novel chemotypes and the extensive use of kinome-wide profiling [20]. 

Chemical proteomics and broad kinome profiling of compound libraries have been implemented as an efficient method to lead to discovery, analyzing targets, and optimization [21]. Results revealed that unknown targets for established drugs presented a viewpoint on the "druggable" kinome, emphasized non-kinase off-targets, and recommended for potential therapeutic applications. A database of the cellular targets of 243 clinical kinase inhibitors has been made available using kinobead technology [21].

The ongoing research will undoubtedly pave the way for a better understanding of molecular pathways that will further unravel the role of PKs in pathogenesis. As of now, the majority of the USFDA-approved PKIs are Protein Tyrosine Kinase inhibitors (PTKIs) followed by protein-serine/threonine PKIs. Most of these drugs are clinically used to treat solid (breast, lung, colon) and non-solid tumors (leukemia). Some PKIs are also effective in treating non-malignant diseases, including myelofibrosis, rheumatoid arthritis, glaucoma, ulcerative colitis, pulmonary fibrosis, etc. [22,23].

## 2. USFDA Approved Protein Kinase Inhibitors

**Figure 1:**
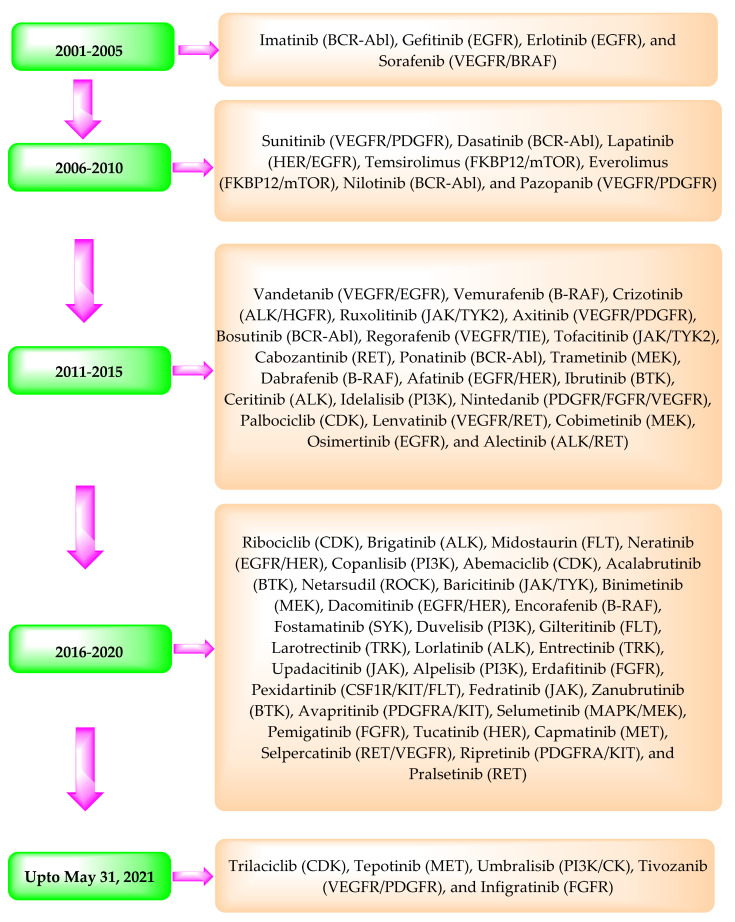
Timeline depicting the approval of the PKIs by the USFDA and their primary targets in brackets.

In 2001, the USFDA approved the marketing of the first clinical PKI, imatinib. Since then, the USFDA has approved about 70 PKIs for clinical use (Table 2) (Figure 1). The data provided in Table 2 have been obtained from USFDA’s Orange Book website (https://www.accessdata.fda.gov/scripts/cder/ob/index.cfm?resetfields=1 (accessed on 31 May 2021) using the drug’s name.

## 3. Patent Searching

The patent searching was performed using the Sci-finder database (CAS Number search, and the exact structure search of each TKI), USFDA’s Orange Book website (mentioned above), and the Drugbank’s website (https://go.drugbank.com/ (accessed on 31 May 2021)) using the drug’s name. The patents disclosing the specific TKI, its marketed active pharmaceutical ingredient, and important polymorphs from the innovative company for the first time were identified and included in this review. The patents of each TKI that claim its treatment methods, dosage forms, formulations, drug combinations, particle size, impurity, preparation process, intermediates, etc., have been excluded from this review. The expiry dates of the selected patents were calculated (20 years from the patent application filing date comprising patent term extension, if any). Sometimes, the drug’s patent term is extended up to five years based on the USPTO’s laws. Accordingly, the expiry dates of the selected patients were also verified from the USPTO’s website. It was also observed that some TKIs were disclosed in different patents of the same patent family and had other expiry dates. In such cases, the patent that had a more extended expiry date was selected for this review because the generic launch of the drug is based on the expiry date of the drug’s patent. The legal status of the patents cited herein was obtained from the website of USPTO (https://portal.uspto.gov/pair/PublicPair (accessed on 31 May 2021)).

## 4. Summary of the Patents

The proprietary name, approved dosage form, approval date, and marketing status of each marketed PKIs are mentioned in Table 2. The patent number, applicant/assignee, expiry date, and legal status of the cited patents of each PKI are provided in Table 3. A brief description of the PKIs and their important patents are provided below.

### 4.1. Imatinib Mesylate

**Figure 2:**
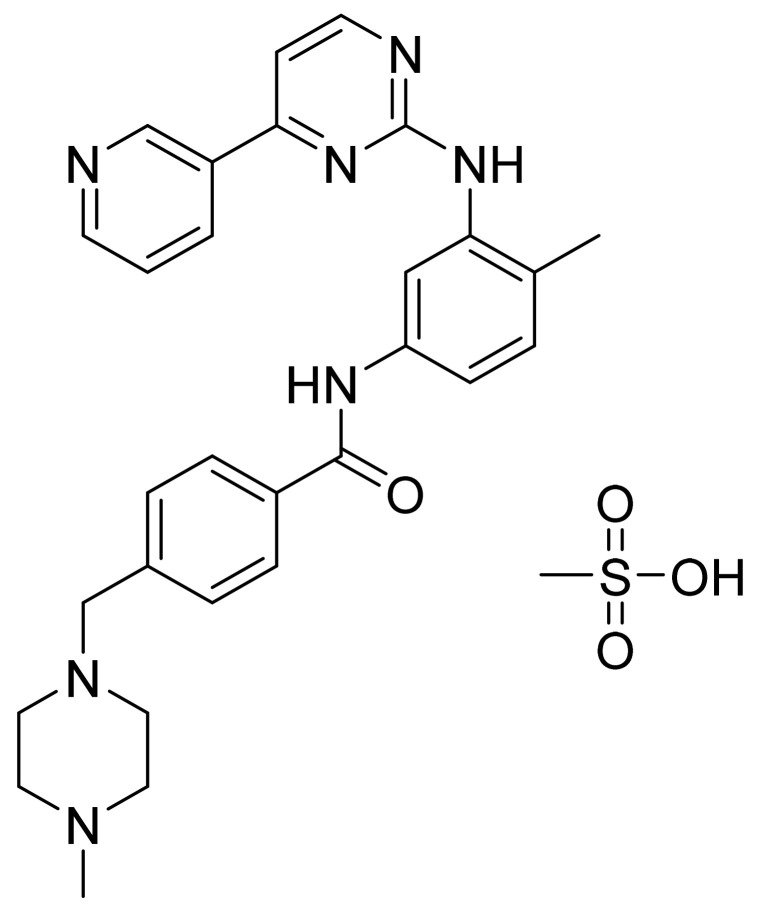
Imatinib mesylate (4-[(4-Methyl-1-piperazinyl)methyl]-*N*-[4-methyl-3-[[4-(3-pyridinyl)-2-pyrimidinyl]amino]phenyl]benzamide methanesulfonate).

Imatinib mesylate (Figure 2) is a pyridine-pyrimidine based piperazine derivative (MF: C_29_H_31_N_7_O·CH_4_SO_3_; MW: 589.7; CAS Number: 220127-57-1) [24]. **US5521184A** claims *N*-phenyl-2-pyrimidine-amine compounds, including imatinib and its pharmaceutically acceptable salts, as antitumor drugs [25]. **USRE43932E** (Re-issue of US7544799B2) claims the β-crystal form of imatinib mesylate as having favorable thermodynamic stability, flow properties, and low hygroscopicity that makes it a suitable active pharmaceutical ingredient (API) to be used in the tablet/capsule dosage forms [26].

### 4.2. Gefitinib

**Figure 3:**
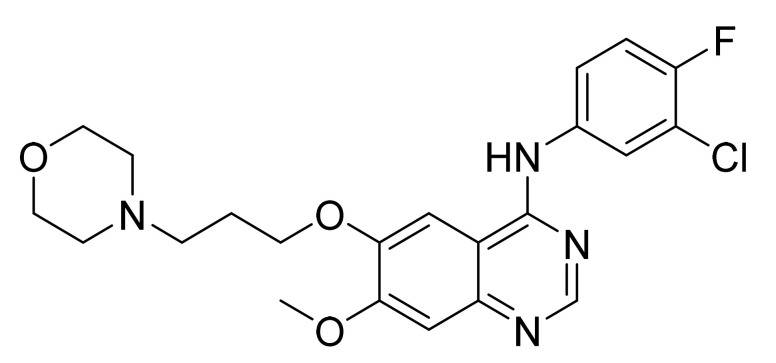
Gefitinib (*N*-(3-chloro-4-fluorophenyl)-7-methoxy-6-[3-(4-morpholinyl)propoxy]-4-quinazolinamine).

Gefitinib (Figure 3) is a morpholine based quinazolinamine derivative (MF: C_22_H_24_ClFN_4_O_3_; MW: 446.9; CAS Number: 184475-35-2) [27]. **US5457105A** unveils quinazoline derivatives and their salts to treat neoplastic disease. This patent claims gefitinib generically [28]. **US5770599A** also covers quinazoline derivatives as anticancer agents. This patent claims gefitinib specifically, along with its pharmaceutically acceptable acid-addition salts [29].

### 4.3. Erlotinib Hydrochloride

**Figure 4:**
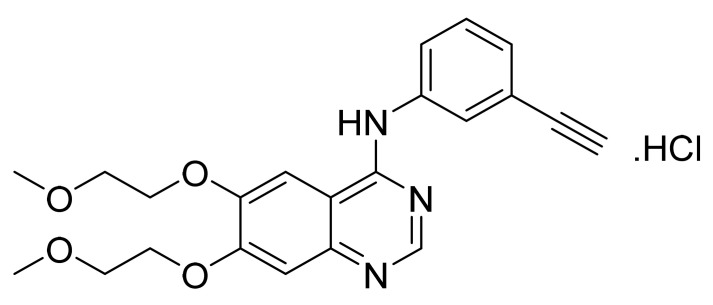
Erlotinib hydrochloride (*N*-(3-ethynylphenyl)-6,7-bis(2-methoxyethoxy)-4-quinazolinamine hydrochloride).

Erlotinib hydrochloride (Figure 4) is a quinazolinamine derivative (MF: C_22_H_23_N_3_O_4_·HCl; MW: 429.90; CAS Number: 183319-69-9) [30]. **USRE41065E** (Reissue patent of US5747498) discloses 4-(substituted phenylamino)quinazoline derivatives, which are useful in treating cancers. It also claims erlotinib hydrochloride specifically [31]. **US6900221B1** provides polymorphs of erlotinib hydrochloride and processes for their selective production. It claims homogeneous thermodynamically stable crystalline polymorph of erlotinib hydrochloride (Form B), suitable for making tablet dosage forms [32].

### 4.4. Sorafenib Tosylate

**Figure 5:**
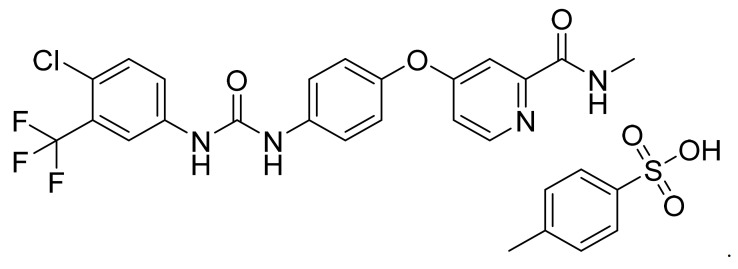
Sorafenib tosylate (4-[4-({[4-chloro-3-(trifluoromethyl)phenyl]carbamoyl}amino)phenoxy]-*N*-methylpyridine-2-carboxamide 4-methylbenzenesulfonate).

Sorafenib tosylate (Figure 5) is a urea-pyridine based diaryl ether derivative (MF: C_21_H_16_ClF_3_N_4_O_3_·C_7_H_8_O_3_S; MW: 637.0; CAS Number: 475207-59-1) [33]. **US7235576B1** provides aryl urea derivatives for treating RAF-mediated diseases like cancer and their pharmaceutical compositions. It claims sorafenib tosylate specifically [34]. **US8877933B2** discloses novel polymorphs of sorafenib tosylate, processes for its synthesis, and compositions comprising it. It claims thermodynamically stable polymorph (Form I) of sorafenib tosylate, which can provide quality dosage form concerning bioavailability and patient safety [35].

### 4.5. Sunitinib Malate

**Figure 6:**
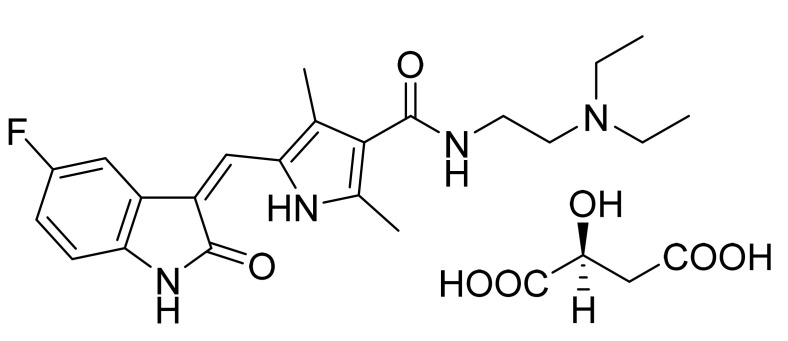
Sunitinib malate (*N*-[2-(diethylamino)ethyl]-5-[(*Z*)-(5-fluoro-1,2-dihydro-2-oxo-3H-indol-3-ylidine)methyl]-2,4-dimethyl-1H-pyrrole-3-carboxamide (2S)-2-hydroxybutanedioic acid).

Sunitinib malate (Figure 6) is an indole based pyrrole-3-carboxamide derivative (MF: C_22_H_27_FN_4_O_2_·C_4_H_6_O_5_; MW: 532.6; CAS Number: 341031-54-7) [36]. **US7125905B2** covers 3-pyrrole substituted 2-indolinone compounds as PK activity modulators for treating disorders related to abnormal PK activity. It claims sunitinib malate specifically [37]. The sunitinib malate is also claimed in **US6573293B2** [38].

### 4.6. Dasatinib Monohydrate

**Figure 7:**
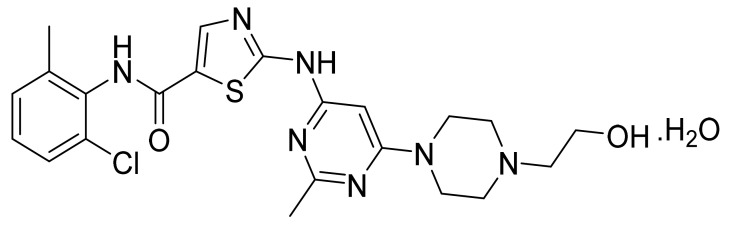
Dasatinib monohydrate (*N*-(2-chloro-6-methylphenyl)-2-[[6-[4-(2-hydroxyethyl)-1-piperazinyl]-2-methyl-4-pyrimidinyl]amino]-5-thiazole carboxamide monohydrate).

Dasatinib monohydrate (Figure 7) is a piperazine-pyrimidine-thiazole based anilide (MF: C_22_H_26_ClN_7_O_2_S·H_2_O; MW: 506.02; CAS Number: 863127-77-9) [39]. **US6596746B1** provides cyclic compounds for use as PKIs to treat cancer. It claims dasatinib specifically [40]. **US7491725B2** claims crystalline monohydrate of dasatinib and process for its preparation [41].

### 4.7. Lapatinib Ditosylate Monohydrate

**Figure 8:**
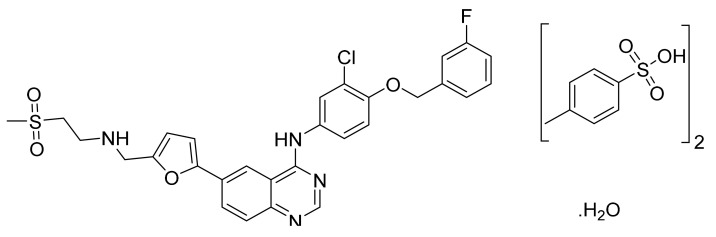
Lapatinib ditosylate monohydrate (*N*-(3-chloro-4-{[(3-fluorophenyl) methyl]oxy}phenyl)-6-[5-({[2-(methylsulfonyl)ethyl]amino}methyl)-2-furanyl]-4-quinazolinamine bis(4-methylbenzenesulfonate) monohydrate).

Lapatinib ditosylate monohydrate (Figure 8) is a furan based quinazolinamine derivative (MF: C_29_H_26_ClFN_4_O_4_S·(C_7_H_8_O_3_S)_2_.H_2_O; MW: 943.5; CAS Number: 388082-78-8) [42]. **US8513262B2** discloses substituted heteroaromatic compounds, their synthesis, compositions, and their use in medicine as PTKIs. It claims lapatinib specifically [43]. **US7157466B2** relates to quinazoline compounds, anhydrate and hydrate ditosylate salts thereof, and the process for their preparation. It claims lapatinib ditosylate monohydrate specifically. The claimed lapatinib ditosylate possesses physical stability and moisture sorption properties superior to di-HCl salt, making it suitable for developing tablet formulations [44].

### 4.8. Temsirolimus

**Figure 9:**
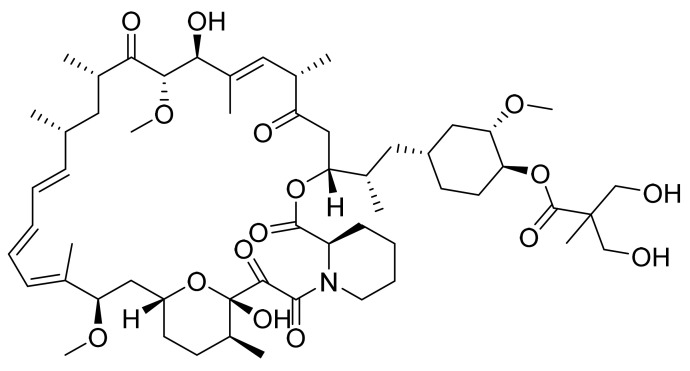
Temsirolimus ((3S,6R,7E,9R,10R,12R,14S,15E,17E,19E,21S,23S,26R,27R,34aS)-9,10,12,13,14,21,22,23,24,25,26,27,32,33,34,34a-Hexadecahydro-9,27-dihydroxy-3-[(1R)-2-[(1S,3R,4R)-4-hydroxy-3-methoxycyclohexyl]-1-methylethyl]-10,21-dimethoxy-6,8,12,14,20,26- hexamethyl-23,27-epoxy-3H-pyrido[2,1-c][1,4]oxaazacyclohentriacontine-1,5,11,28,29(4H,6H,31H)-pentone 4′-[2,2-bis(hydroxymethyl)propionate]).

Temsirolimus (Figure 9) is a piperidine-tetrahydropyran based macrolide lactams (MF: C_56_H_87_NO_16_; MW: 1030.30; CAS Number: 162635-04-3) [45]. **USRE44768E** (Reissue of US5362718) relates to hydroxy esters of rapamycin for treating T-cell leukemia/lymphoma, solid tumors, and hyperproliferative vascular disorders. It claims temsirolimus specifically [46].

### 4.9. Everolimus

**Figure 10:**
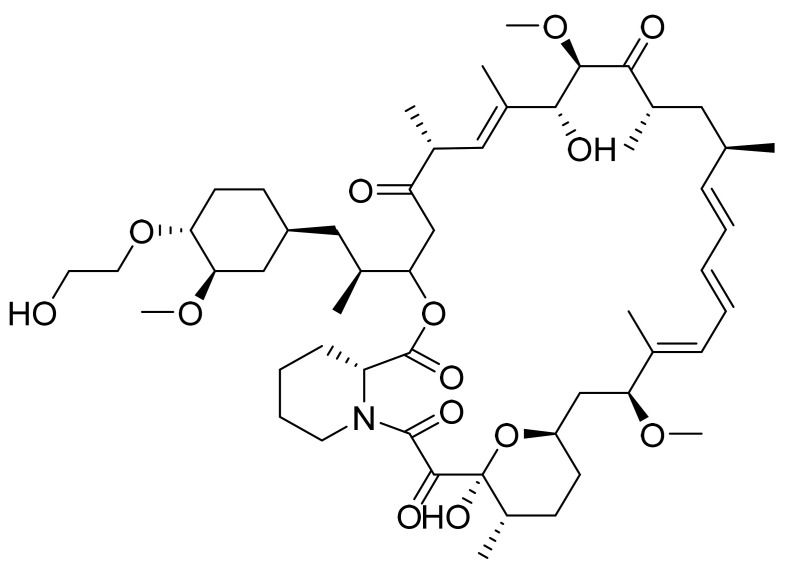
Everolimus ((1R,9S,12S,15R,16E,18R,19R,21R,23S,24E,26E,28E,30S,32S,35R)-1,18-dihydroxy-12-{(1R)-2-[(1S,3R,4R)-4-(2-hydroxyethoxy)-3-methoxycyclohexyl]-1-methylethyl}-19,30-dimethoxy-15,17,21,23,29,35-hexamethyl-11,36-dioxa-4-aza-tricyclo[30.3.1.04,9]hexatriaconta-16,24,26,28-tetraene-2,3,10,14,20-pentaone).

Everolimus (Figure 10) is a piperidine-tetrahydropyran based macrolide lactam (MF: C_53_H_83_NO_14_; MW: 958.25; CAS Number: 159351-69-6) [47]. **US5665772A** provides alkylated derivatives of rapamycin as immunosuppressants. It claims everolimus specifically [48].

### 4.10. Nilotinib Hydrochloride Monohydrate

**Figure 11:**
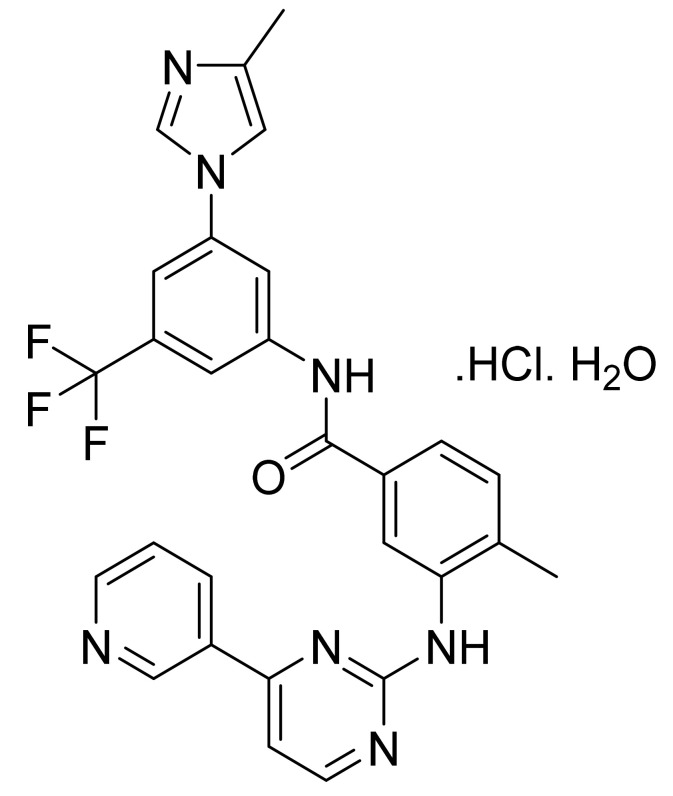
Nilotinib hydrochloride monohydrate (4-methyl-*N*-[3-(4-methyl-1H-imidazol-1-yl)-5-(trifluoromethyl)phenyl]-3-[[4-(3-pyridinyl)-2-pyrimidinyl]amino]-benzamide monohydrochloride monohydrate).

Nilotinib hydrochloride monohydrate (Figure 11) is a pyridine-pyrimidine-imidazole-based benzanilide derivative (MF: C_28_H_22_F_3_N_7_O·HCl·H_2_O; MW: 584; CAS Number: 923288-90-8) [49]. **US7169791B2** covers substituted pyrimidinyl aminobenzamides, methods of synthesis, and their compositions to treat neoplastic diseases like leukemia. It claims nilotinib and its salts [50]. **US8163904B2** claims nilotinib hydrochloride monohydrate as having physicochemical properties required to develop a good dosage form [51]. **US8415363B2** claims crystalline form B of nilotinib hydrochloride monohydrate having superior crystallinity and physical stability over other polymorphs [52].

### 4.11. Pazopanib Hydrochloride

**Figure 12:**
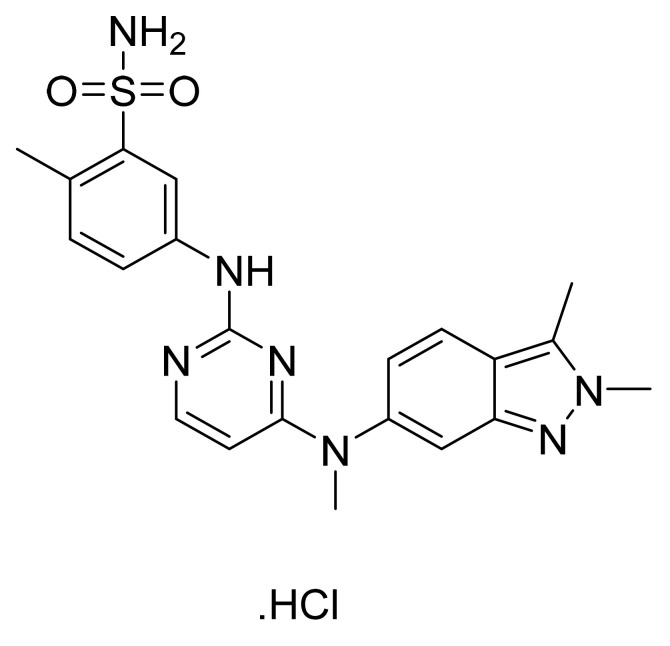
Pazopanib hydrochloride (5-[[4-[(2,3-*d*imethyl-2H-indazol-6-yl)methylamino]-2-pyrimidinyl]amino]-2-methylbenzenesulfonamide monohydrochloride).

Pazopanib hydrochloride (Figure 12) is a benzenesulfonamide bearing benzimidazole-pyrimidinyl compound (MF: C_21_H_23_N_7_O_2_S·HCl; MW: 473.99; CAS Number: 635702-64-6) [53]. **US7105530B2** reports pyrimidine derivatives as inhibitors of VEGFR-2 to treat disorders, including cancer, associated with inappropriate angiogenesis. It claims pazopanib and its salts [54]. **US8114885B2** claims pazopanib hydrochloride precisely [55]. The claimed hydrochloride salt possesses advantageous properties like stability and solubility to develop quality dosage forms.

### 4.12. Vandetanib

**Figure 13:**
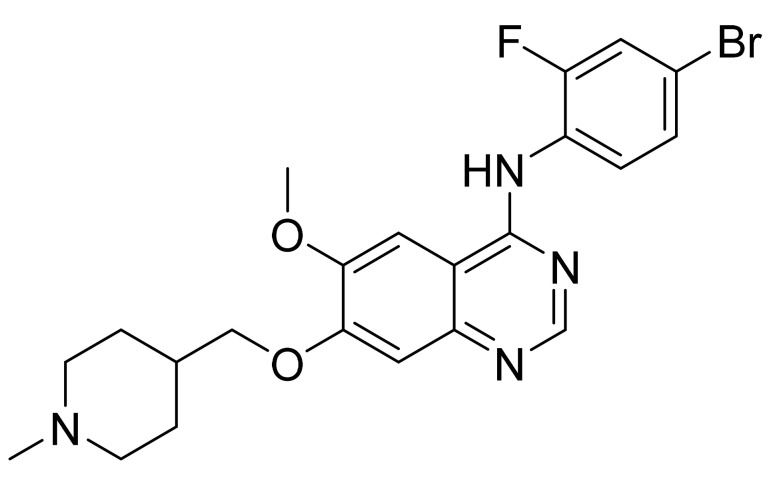
Vandetanib (*N*-(4-bromo-2-fluorophenyl)-6-methoxy-7-[(1-methylpiperidin-4-yl)methoxy]quinazolin-4-amine).

Vandetanib (Figure 13) is a piperidine based 4-aminoquinazolinamine derivative (MF: C_22_H_24_BrFN_4_O_2_; MW: 475.36; CAS Number: 443913-73-3) [56]. **USRE42353E** (Reissue of US6414148B1) provides quinazoline derivatives, synthesis, and compositions to treat illness linked with angiogenesis and amplified vascular permeability. It claims vandetanib precisely [57].

### 4.13. Vemurafenib

**Figure 14:**
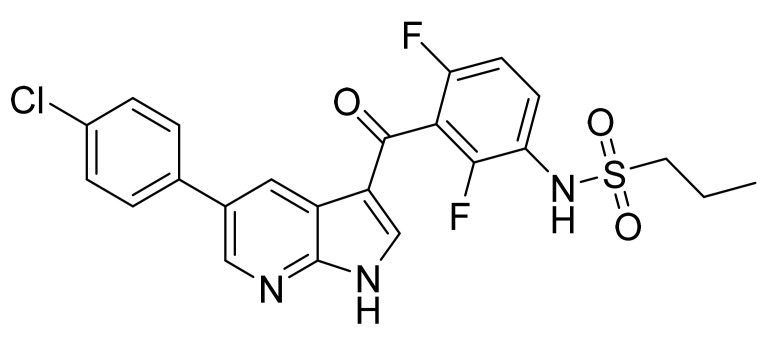
Vemurafenib (Propane-1-sulfonic acid {3-[5-(4-chlorophenyl)-1H-pyrrolo[2,3-*b*]pyridine-3-carbonyl]-2,4-difluoro-phenyl}-amide).

Vemurafenib (Figure 14) is a phenylketone based pyrrolopyridine (MF: C_23_H_18_ClF_2_N_3_O_3_S; MW: 489.9; CAS Number: 918504-65-1) [58]. **US8143271B2** describes pyrrolopyridine based compounds as PTKIs to treat diseases and conditions associated with aberrant activity of PTKs. It claims vemurafenib specifically [59].

### 4.14. Crizotinib

**Figure 15:**
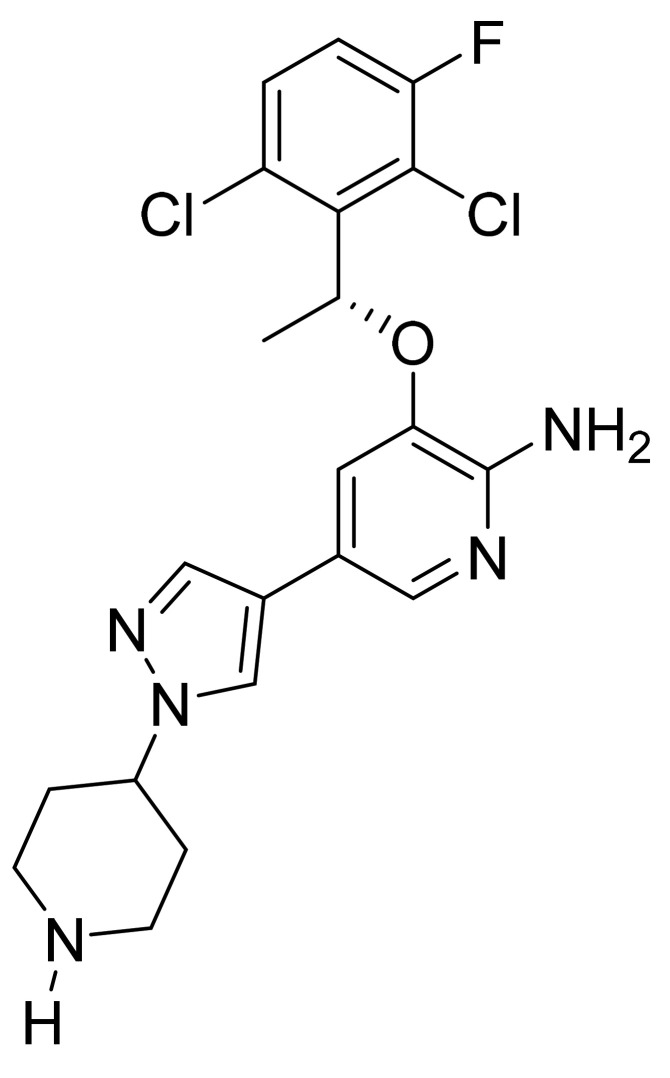
Crizotinib ((R)-3-[1-(2,6-Dichloro-3-fluorophenyl)ethoxy]-5-[1-(piperidin-4-yl)-1H-pyrazol-4-yl]pyridin-2-amine).

Crizotinib (Figure 15) is a piperidine based pyrazolylpyridine derivative (MF: C_21_H_22_Cl_2_FN_5_O; MW: 450.34; CAS Number: 877399-52-5) [60]. **US7858643B2** describes aminopyridines and aminopyrazines having PTKI activity, methods of synthesizing and using these compounds as anticancer agents. It claims crizotinib and its salts [61]. **US8217057B2** claims a crystalline form of a free base of crizotinib with improved solubility, stability, and physicochemical properties to develop solid dosage forms, such as capsules [62].

### 4.15. Ruxolitinib Phosphate

**Figure 16:**
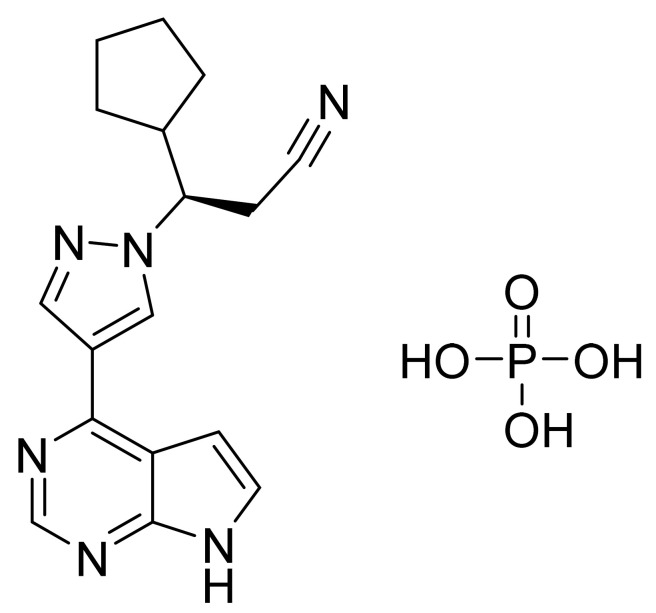
Ruxolitinib phosphate ((R)-3-(4-(7H-pyrrolo[2,3-*d*]pyrimidin-4-yl)-1H-pyrazol-1-yl)-3-cyclopentylpropanenitrile phosphate).

Ruxolitinib phosphate (Figure 16) is a pyrrolo[2,3-*d*]pyrimidine based pyrazole derivative (MF: C_17_H_21_N_6_O_4_P; MW: 404.36; CAS Number: 1092939-17-7) [63]. **US7598257B2** provides pyrrolo[2,3-*b*]pyridines as JAK modulators, which are beneficial to treat immune-related disorders, skin diseases, myeloid proliferative ailments, and cancer. It claims ruxolitinib and its salts [64]. **US8722693B2** claims ruxolitinib phosphate, which has improved water solubility, dissolution rate, chemical stability, long shelf life, excipients, and reproducibility compared to the free base [65].

### 4.16. Axitinib

**Figure 17:**
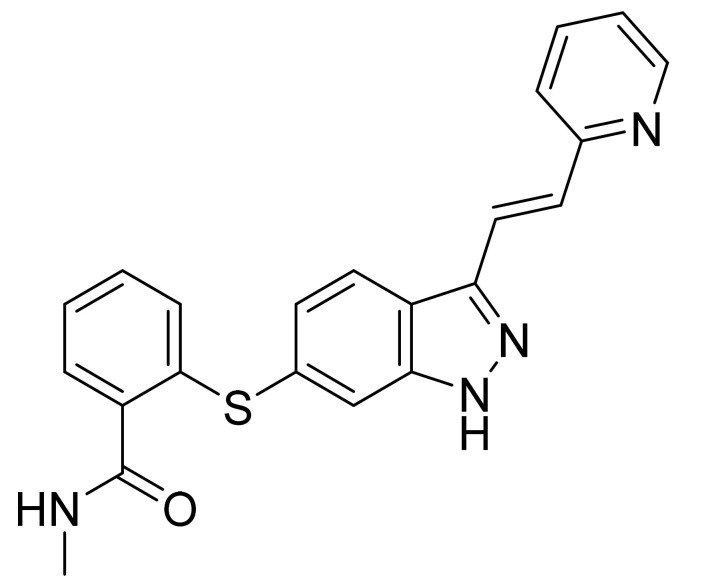
Axitinib (*N*-methyl-2-[3-((*E*)-2-pyridin-2-yl-vinyl)-1H-indazol-6-ylsulfanyl]-benzamide).

Axitinib (Figure 17) is a pyridine based indazolylphenyl thioether (MF: C_22_H_18_N_4_OS; MW: 386.47; CAS Number: 319460-85-0) [66]. **US6534524B1** relates to indazole compounds as PTKIs and their pharmaceutical compositions to treat diseases linked with undesirable angiogenesis and cellular proliferation. It claims axitinib specifically [67]. **US8791140B2** claims crystalline forms of axitinib that have advantages in bioavailability, stability, manufacture ability, and suitability for bulk preparation [68].

### 4.17. Bosutinib Monohydrate

**Figure 18:**
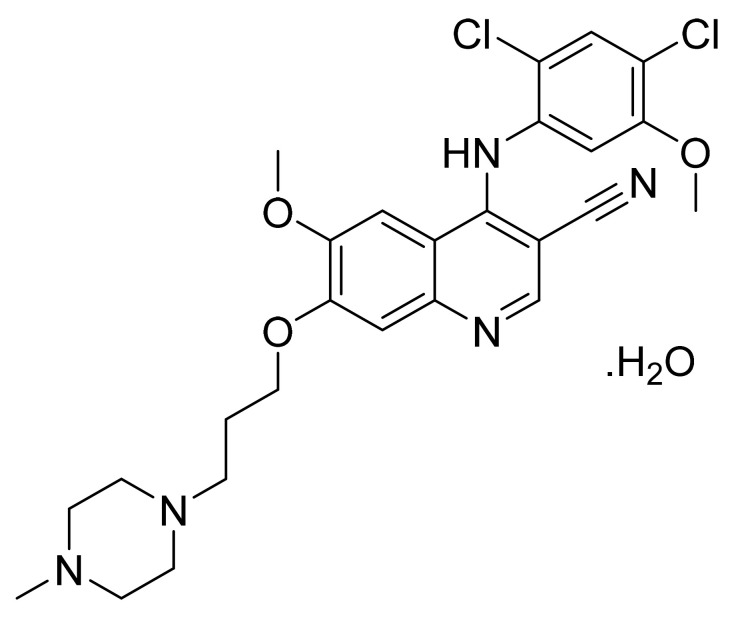
Bosutinib monohydrate (4-[(2,4-dichloro-5-methoxyphenyl)amino]-6-methoxy-7-[3-(4-methylpiperazin-1-yl)propoxy]quinoline-3-carbonitrile monohydrate).

Bosutinib monohydrate (Figure 18) is a piperazine based 3-quinolinecarbonitrile derivative (MF: C_26_H_29_Cl_2_N_5_O_3_·H_2_O; MW: 548.46; CAS Number: 918639-08-4) [69]. **USRE42376E** (Reissue of US6297258B1) describes substituted 3-cyano quinoline compounds as PTKIs to treat diseases resulting from deregulation of PTKs, for example, cancer and polycystic kidney disease. It claims bosutinib [70]. **US7767678B2** claims non-hygroscopic and stable crystalline bosutinib monohydrate (Form I) having good solubility that can be used to prepare different solid dosage forms [71].

### 4.18. Regorafenib Monohydrate

**Figure 19:**
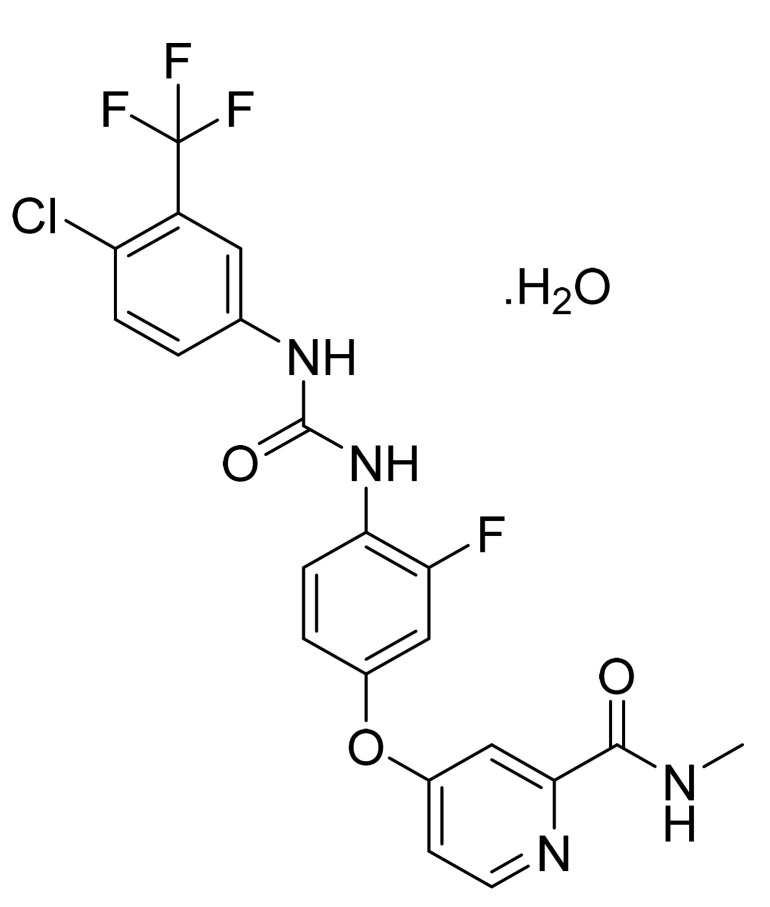
Regorafenib monohydrate (4-[4-({[4-chloro-3-(trifluoromethyl)phenyl] carbamoyl}amino)-3-fluorophenoxy]-*N*-methylpyridine-2-carboxamide monohydrate).

Regorafenib monohydrate (Figure 19) is pyridinylphenyl urea derivative (MF: C_21_H_15_ClF_4_N_4_O_3_·H_2_O; MW: 500.83; CAS Number: 1019206-88-2) [72]. **US8637553B2** discloses omega-carboxyaryl diphenyl urea derivatives as potent inhibitors of PDGFR, VEGFR, RAF, and p38 kinase to treat cancer, inflammatory diseases, and osteoporosis. It claims regorafenib and its salts [73]. **US9957232B2** claims regorafenib monohydrate with high stability and good physicochemical features to manufacture pharmaceutical compositions [74].

### 4.19. Tofacitinib Citrate

**Figure 20:**
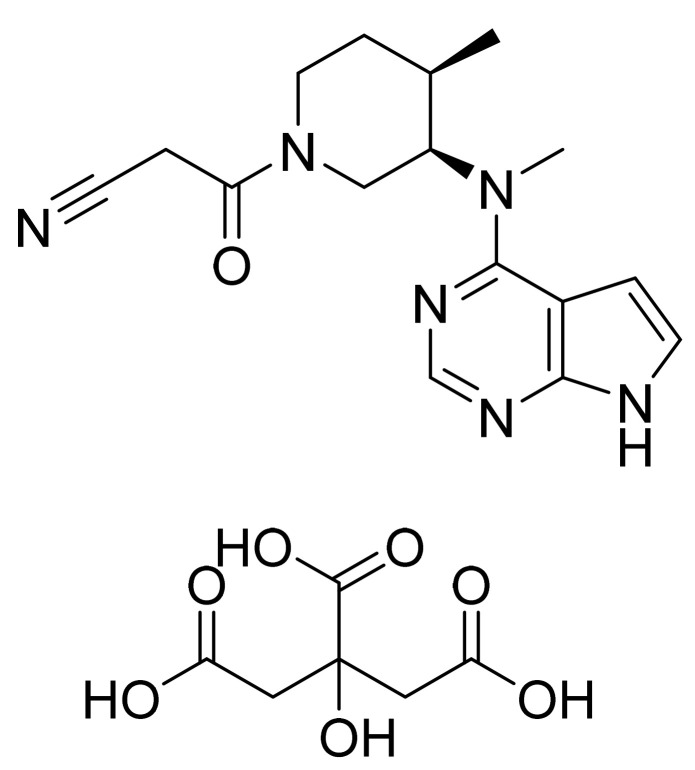
Tofacitinib citrate ((3R,4R)-4-methyl-3-(methyl-7H-pyrrolo[2,3-*d*]pyrimidin-4-ylamino)-ß-oxo-1-piperidinepropanenitrile 2-hydroxy-1,2,3-propanetricarboxylate (1:1)).

Tofacitinib citrate (Figure 20) is an pyrrolo[2,3-*d*]pyrimidine based piperidine derivative (MF: C_16_H_20_N_6_O·C_6_H_8_O_7_; MW: 504.5; CAS Number: 540737-29-9) [75]. **USRE41783E** (Reissue of US6627754B2) provides pyrrolo[2,3-*d*]pyrimidines as JAK3 inhibitors to treat rheumatoid arthritis, psoriasis, cancer, and leukemia. It claims tofacitinib and its salt [76]. **US6965027B2** claims a crystalline form of tofacitinib mono citrate salt with solid-state properties (solubility, stability, compressibility, etc.), which are acceptable to support tablet development [77].

### 4.20. Cabozantinib S-Malate

**Figure 21:**
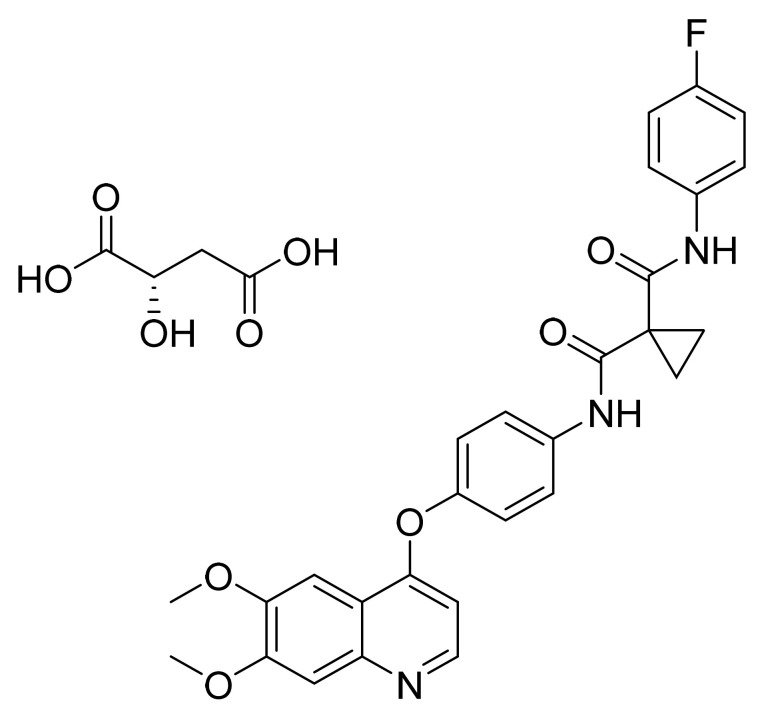
Cabozantinib (S)-malate (*N*-(4-(6,7-dimethoxyquinolin-4-yloxy)phenyl)-*N′*-(4-fluorophenyl)cyclopropane-1,1-dicarboxamid (2S)-hydroxybutanedioate).

Cabozantinib S-malate (Figure 21) is a quinolinylphenyl ether derivative (MF: C_28_H_24_FN_3_O_5_·C_4_H_6_O_5_; MW: 635.6; CAS Number: 1140909-48-3) [78]. **US7579473B2** relates to quinazolines and quinolines as TKIs, and their pharmaceutical compositions to treat psoriasis, multiple sclerosis, and rheumatoid arthritis. It claims cabozantinib and its salts [79]. **US8877776B2** claims cabozantinib (L)-malate salt having desirable solubility and chemical/physical stability to develop a tablet/capsule dosage forms for intended use [80].

### 4.21. Ponatinib Hydrochloride

**Figure 22:**
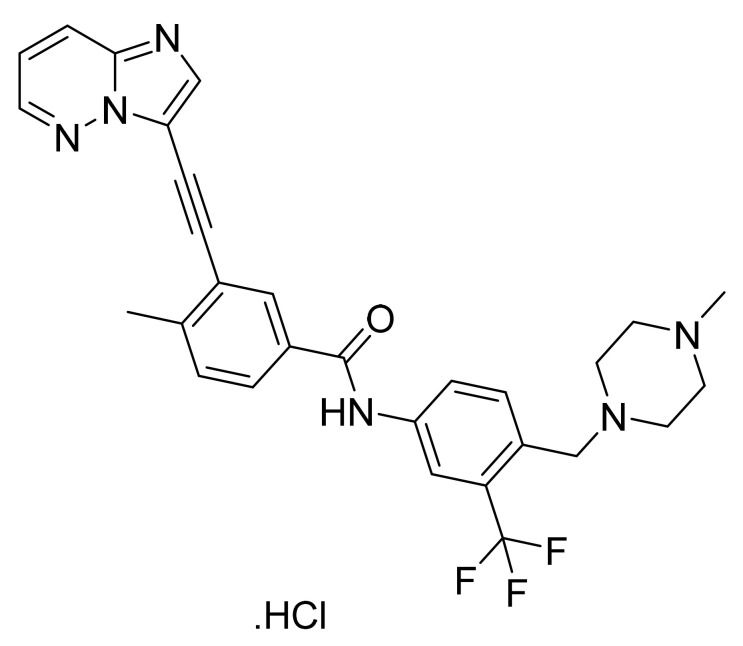
Ponatinib hydrochloride (3-(imidazo[1,2-*b*]pyridazin-3-ylethynyl)-4-methyl-*N*-{4-[(4-methylpiperazin-1-yl)methyl]-3-(trifluoromethyl)phenyl}benzamide hydrochloride).

Ponatinib hydrochloride (Figure 22) is animidazo[1,2-*b*]pyridazine based piperazine derivative (MF: C_29_H_28_ClF_3_N_6_O; MW: 569.02; CAS Number: 1114544-31-8) [81]. **US8114874B2** describes imidazo[1,2-*b*]pyridazines as PTKIs and their pharmaceutical compositions to treat cancer and other diseases mediated by PTKs. It claims ponatinib hydrochloride specifically [82]. **US9493470B2** claims stable crystalline form A of ponatinib hydrochloride that is advantageous for the commercial preparation of solid dosage forms because of its physicochemical stability compared to amorphous ponatinib hydrochloride [83].

### 4.22. Trametinib Dimethyl Sulfoxide

**Figure 23:**
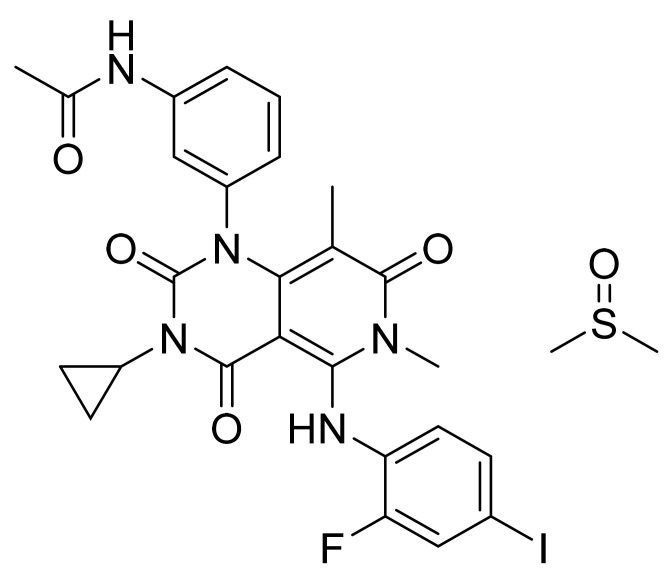
Trametinib dimethyl sulfoxide (*N*-(3-{3-cyclopropyl-5-[(2-fluoro-4-iodophenyl)amino]-6,8-dimethyl-2,4,7-trioxo-1H,2H,3H,4H,6H,7H-pyrido[4,3-*d*]pyrimidin-1-yl}phenyl)acetamide dimethyl sulfoxide).

Trametinib dimethyl sulfoxide (Figure 23) is a pyridopyrimidine derivative (MF: C_26_H_23_FIN_5_O_4_.C_2_H_6_OS; MW: 693.53; CAS Number: 1187431-43-1) [84]. **US7378423B2** unveils pyrimidine compounds, their salts, synthetic procedures, and compositions to treat ailments caused by unwanted cell proliferation, for example, cancer. It claims trametinib dimethyl sulfoxide specifically [85].

### 4.23. Dabrafenib Mesylate

**Figure 24:**
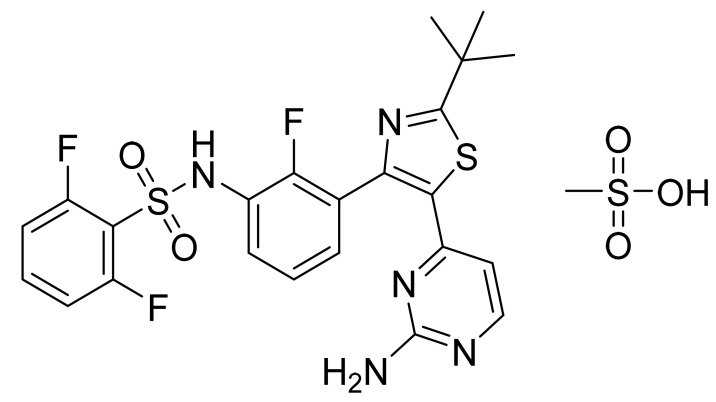
Dabrafenib mesylate (*N*-{3-[5-(2-amino-4-pyrimidinyl)-2-(1,1-dimethylethyl)-1,3-thiazol-4-yl]-2-fluorophenyl}-2,6-difluorobenzene sulfonamide mesylate).

Dabrafenib mesylate (Figure 24) is a pyrimidine-thiazole based diphenyl sulfonamide derivative (MF: C_23_H_20_F_3_N_5_O_2_S_2_.CH_4_O_3_S; MW: 615.68; CAS Number: 1195768-06-9) [86]. **US7994185B2** provides benzene sulfonamide thiazole and oxazole compounds, their pharmaceutical compositions, processes for their preparation, and methods of using these compounds and compositions for treating cancer and melanoma. It claims dabrafenib mesylate specifically [87].

### 4.24. Afatinib Dimaleate

**Figure 25:**
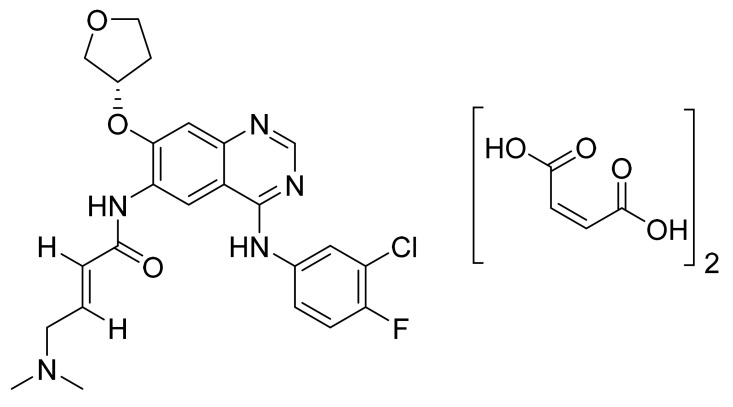
Afatinib dimaleate (*N*-[4-[(3-chloro-4-fluorophenyl)amino]-7-[[(3S)-tetrahydro-3-furanyl]oxy]-6-quinazolinyl]-4-(dimethylamino)but-2-enamide dimaleate).

Afatinib dimaleate (Figure 25) is a tetrahydrofuran based quinazolinamine derivative (MF: C_32_H_33_ClFN_5_O_11_; MW: 718.1; CAS Number: 850140-73-7) [88]. **USRE43431E** (Reissue of US7019012B2) unveils quinazoline derivatives and their physiologically acceptable salts possessing an inhibitory effect on signal transduction mediated by PTKs to treat tumoral diseases, diseases of the lungs, and respiratory tract. It claims afatinib dimaleate precisely [89]. **US8426586B2** claims crystalline afatinib dimaleate, synthesis, and its compositions. The claimed crystalline form is stable and has advantageous properties to develop quality dosage forms [90].

### 4.25. Ibrutinib

**Figure 26:**
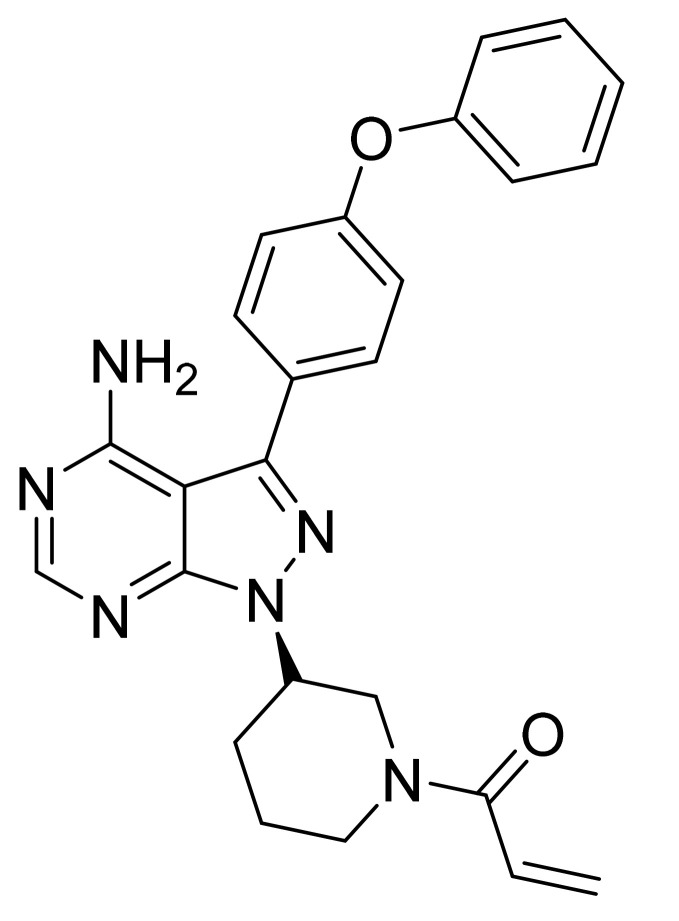
Ibrutinib (1-[(3R)-3-[4-amino-3-(4-phenoxyphenyl)-1H-pyrazolo[3,4-*d*]pyrimidin-1-yl]-1-piperidinyl]-2-propen-1-one).

Ibrutinib (Figure 26) is a piperidine based pyrazolo[3,4-*d*]pyrimidine (MF: C_25_H_24_N_6_O_2_; MW: 440.50; CAS Number: 936563-96-1) [91]. **US8735403B2** describes pyrazolo[3,4-*d*]pyrimidine based inhibitors of BTK, their synthesis, and compositions to treat diseases, wherein inhibition of BTK delivers therapeutic advantage to the diseased person. It claims ibrutinib specifically [92]. **US9296753B2** claims stable, water-soluble, and non-hygroscopic crystalline ibrutinib that can be used to manufacture quality dosage forms [93].

### 4.26. Ceritinib

**Figure 27:**
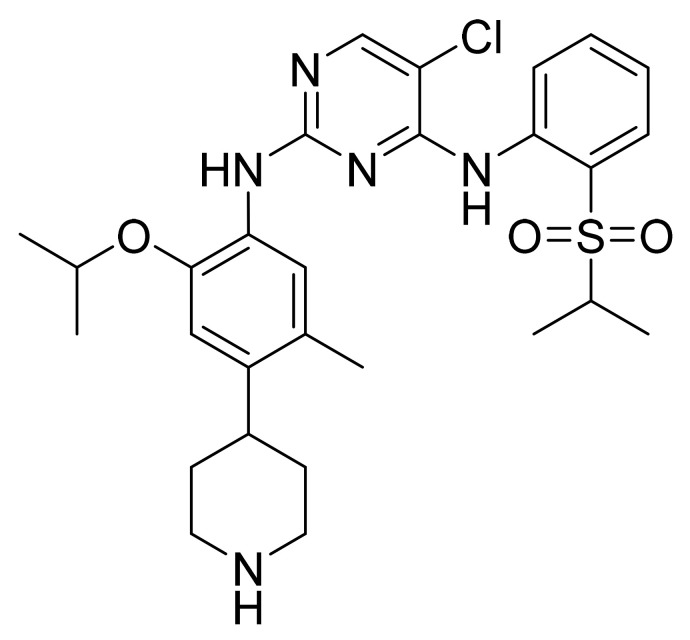
Ceritinib (5-Chloro-*N*4-[2-[(1-methylethyl)sulfonyl]phenyl]-*N*2-[5-methyl-2-(1-methylethoxy)-4-(4-piperidinyl)phenyl]-2,4-pyrimidinediamine).

Ceritinib (Figure 27) is a pyrimidine based phenylpiperidine derivative (MF: C_28_H_36_N_5_O_3_ClS; MW: 558.14; CAS Number: 1032900-25-6) [94]. **US8039479B2** reveals pyrimidine and pyridine derivatives and their pharmaceutical compositions to treat a condition that responds to inhibition of ALK, FAK, ZAP-70, IGF-1R, or a combination thereof. It claims ceritinib specifically [95]. **US9309229B2** claims a pure and stable crystalline form of ceritinib with desirable physicochemical properties to provide good dosage forms [96].

### 4.27. Idelalisib

**Figure 28:**
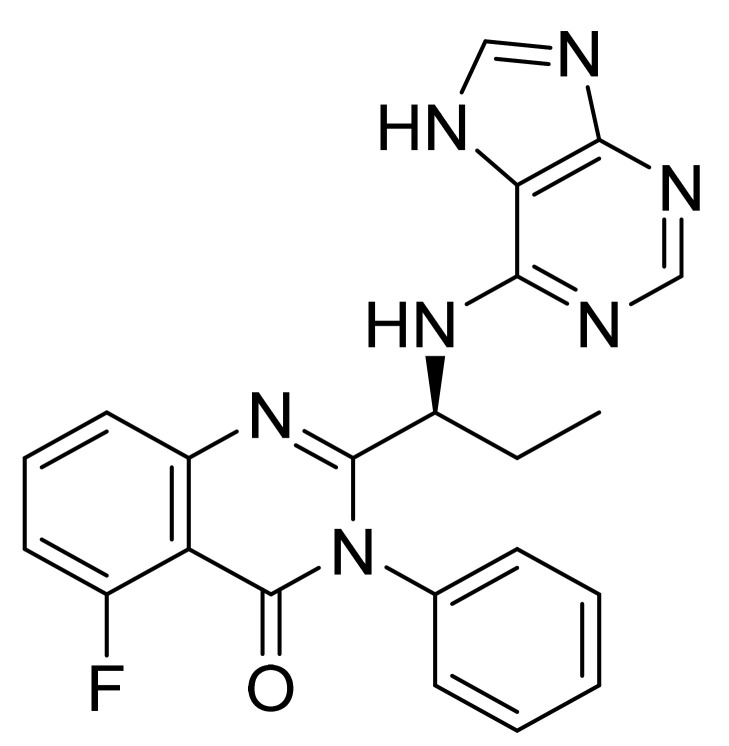
Idelalisib (5-fluoro-3-phenyl-2-[(1S)-1-(9H-purin-6-ylamino)propyl]quinazolin-4(3H)-one).

Idelalisib (Figure 28) is a purine based quinazolinone derivative (MF: C_22_H_18_FN_7_O; MW: 415.42; CAS Number: 870281-82-6) [97]. **USRE44638E** (Reissue of US7932260B2) reports substituted quinazolinone compounds as PI3K_δ_ inhibitors to treat diseases like bone-resorption disorders, hematopoietic cancers, lymphomas, multiple myelomas, and leukemia. It claims idelalisib and its salts [98]. **US9469643B2** claims a water-soluble bioavailable and stable polymorph of idelalisib (Form II) that can be used to provide quality dosage forms [99].

### 4.28. Nintedanib Esylate

**Figure 29:**
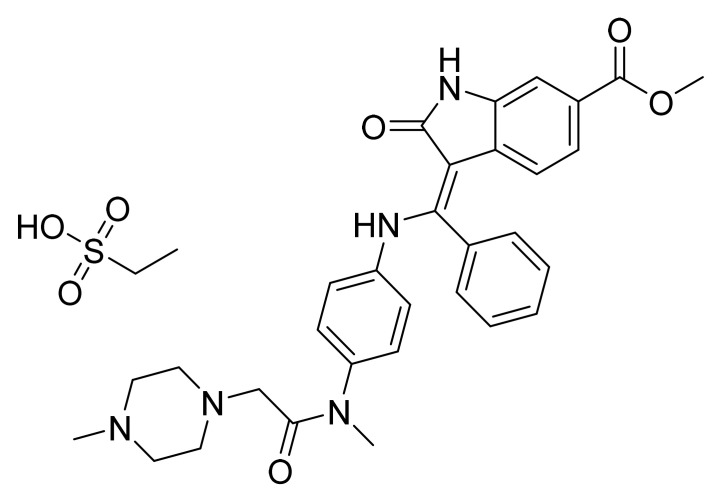
Nintedanib esylate (methyl (3Z)-3-[({4-[*N*-methyl-2-(4-methylpiperazin-1-yl)acetamido]phenyl}amino)(phenyl)methylidene]-2-oxo-2,3-dihydro-1H-indole-6-carboxylate esylate).

Nintedanib esylate (Figure 29) is a piperazine based indole carboxylic acid derivative (MF: C_31_H_33_N_5_O_4_.C_2_H_6_O_3_S; MW: 649.76; CAS Number: 656247-18-6) [100]. **US6762180B1** states indolinone derivatives as PTKIs, synthesis, and compositions to treat proliferative sicknesses. It claims nintedanib and its salts [101]. **US7119093B2** claims a stable nintedanib esylate salt specifically characterized by good crystallinity and low amorphization during grinding and compression. This salt is claimed to have good physicochemical characteristics to support quality dosage forms [102].

### 4.29. Palbociclib

**Figure 30:**
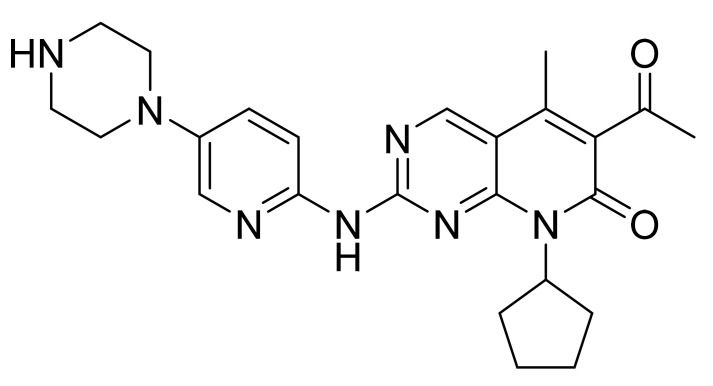
Palbociclib (6-acetyl-8-cyclopentyl-5-methyl-2-{[5-(piperazin-1-yl)pyridin-2-yl]amino}pyrido[2,3-*d*]pyrimidin-7(8H)-one).

Palbociclib (Figure 30) is a pyrido[2,3-*d*]pyrimidine based pyridinylpiperazine derivative (MF: C_24_H_29_N_7_O_2_; MW: 447.54; CAS: 571190-30-2) [103]. **USRE47739E** (Reissue of US7208489B2) delivers substituted 2-amino pyridines as potent inhibitors of CDK 4, useful for treating inflammation and proliferative cell diseases such as cancer and restenosis. It claims palbociclib and its salts [104]. **US10723730B2** claims a stable crystalline free base of palbociclib with larger primary particle size, reduced specific surface area, lower surface energy measurements, and physicochemical properties to formulate a good dosage form [105].

### 4.30. Lenvatinib Mesylate

**Figure 31:**
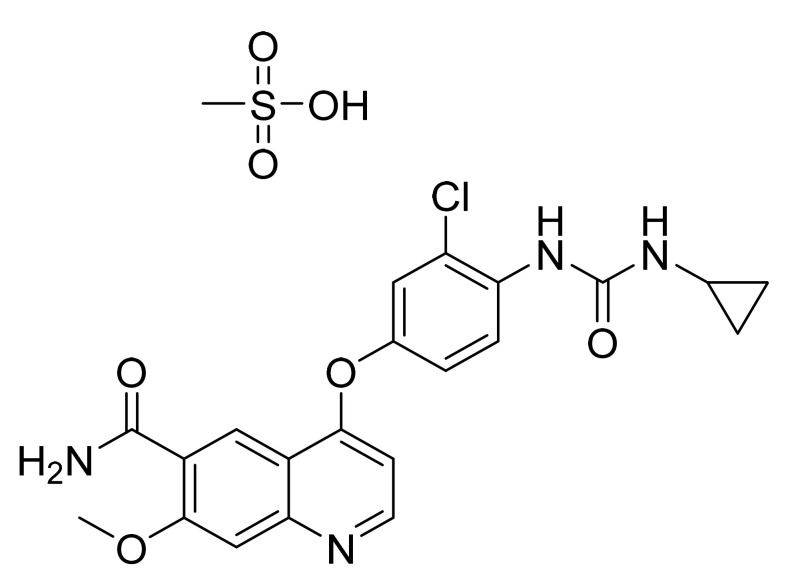
Lenvatinib mesylate (4-[3-chloro-4-(*N′*-cyclopropylureido)phenoxy]-7-methoxyquinoline-6-carboxamide methanesulfonate).

Lenvatinib mesylate (Figure 31) is a quinoline carboxamide derivative (MF: C_21_H_19_ClN_4_O_4_.CH_4_O_3_S; MW: 522.96; CAS Number: 857890-39-2) [106]. **US7253286B2** reports nitrogen-containing aromatic derivatives and salts or hydrates thereof to treat various diseases associated with abnormal angiogenesis. It claims lenvatinib and its pharmacologically active salts [107]. **US7612208B2** claims a crystalline form of lenvatinib mesylate with improved features (physical/pharmacokinetics) compared to the free-form [108].

### 4.31. Cobimetinib Fumarate

**Figure 32:**
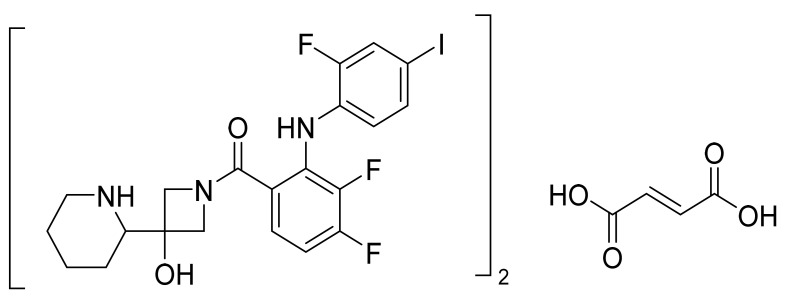
Cobimetinib fumarate ((S)-[3,4-difluoro-2-(2-fluoro-4-iodophenylamino)phenyl][3-hydroxy-3-(piperidin-2-yl)azetidin-1-yl]methanone hemifumarate).

Cobimetinib fumarate (Figure 32) is a piperidine-azetidine based anthranilamide derivative (MF: C_46_H_46_F_6_I_2_N_6_O_8_ (2C_21_H_21_F_3_IN_3_O_2_.C_4_H_4_O_4_); MW: 1178.71; CAS Number: 1369665-02-0) [109]. **US7803839B2** provides azetidin-1-yl(2-(2-fluorophenylamino)cyclic)methanone derivatives as inhibitors of MEK that are useful in cancer treatment. It claims cobimetinib and its salts [110]. **US10590102B2** claims a thermodynamically stable and non-hygroscopic crystalline fumarate salt (Form A) of cobimetinib with suitable properties for use in a pharmaceutical composition [111].

### 4.32. Osimertinib Mesylate

**Figure 33:**
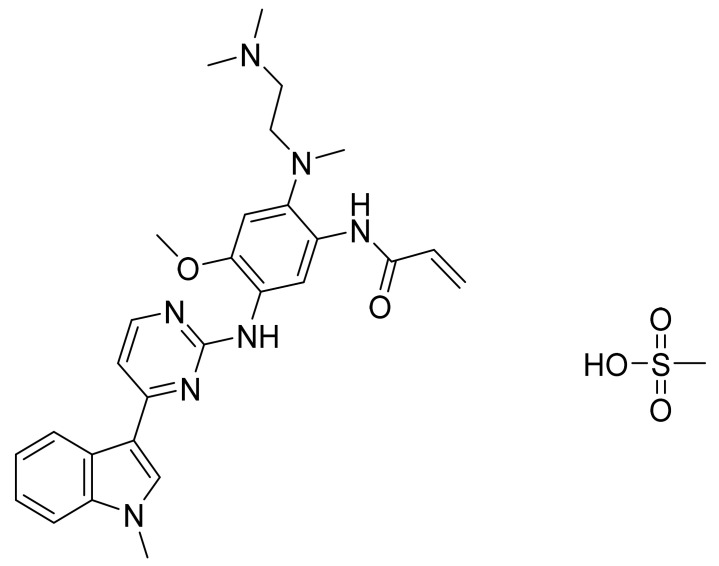
Osimertinib mesylate (*N*-(2-{2-dimethylaminoethyl-methylamino}-4-methoxy-5-{[4-(1-methylindol-3-yl)pyrimidin-2-yl]amino}phenyl)prop-2-enamide mesylate).

Osimertinib mesylate (Figure 33) is a pyrimidine based indole derivative (MF: C_28_H_33_N_7_O_2_.CH_4_O_3_S; MW: 596; CAS Number: 1421373-66-1) [112]. **US8946235B2** states 2-(2,4,5-substituted-anilino)pyrimidines, useful in treating a disease mediated by EGFR, for example, cancer. It claims osimertinib mesylate specifically [113].

### 4.33. Alectinib Hydrochloride

**Figure 34:**
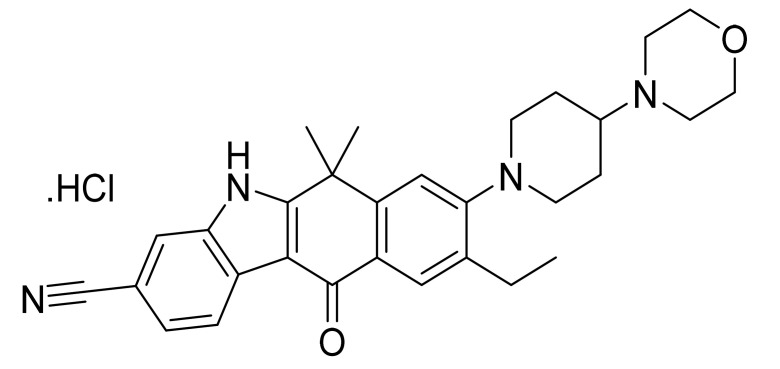
Alectinib hydrochloride (9-Ethyl-6,6-dimethyl-8-[4-(morpholin-4-yl)piperidin-1-yl]-11-oxo-6,11-dihydro-5H-benzo[*b*]carbazole-3-carbonitrile hydrochloride).

Alectinib hydrochloride (Figure 34) is a morpholine-piperidine based carbazole derivatives (MF: C_30_H_34_N_4_O_2_·HCl; MW: 519.08; CAS Number: 1256589-74-8) [114]. **US9126931B2** relates to tetracyclic compounds as ALK inhibitors for treating a disease accompanied by an abnormality in ALK, for example, cancer, depression, and cognitive function disorder. It claims alectinib and its salts [115].

### 4.34. Ribociclib Succinate

**Figure 35:**
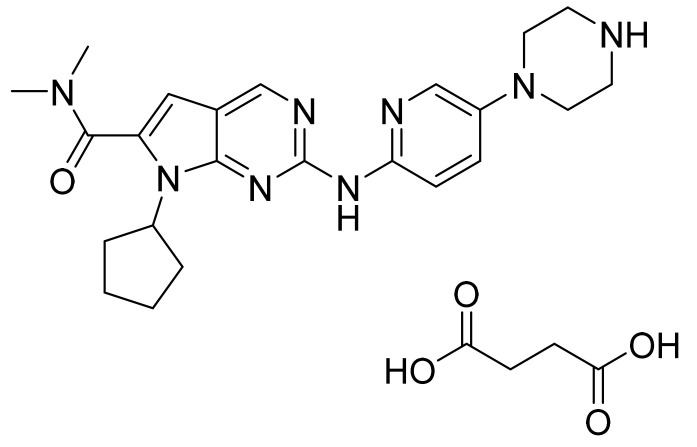
Ribociclib succinate (7-cyclopentyl-*N,N*-dimethyl-2-{[5-(piperazin-1-yl)pyridin-2-yl]amino}-7H-pyrrolo[2,3-*d*]pyrimidine-6-carboxamide succinate).

Ribociclib succinate (Figure 35) is a pyridine-piperazine based pyrrolo[2,3-*d*]pyrimidine derivative (MF: C_23_H_30_N_8_O·C_4_H_6_O_4_; MW: 552.64; CAS Number: 1374639-75-4) [116]. **US8415355B2** discloses pyrrolopyrimidine compounds, the process for their preparation, and their pharmaceutical compositions to treat a disease linked with CDK 4 inhibition. It claims ribociclib and its salts [117]. **US9193732B2** claims succinate salt of ribociclib that has good stability, non-hygroscopicity, and good solubility. These features make this salt a suitable salt to develop the desired formulation [118].

### 4.35. Brigatinib

**Figure 36:**
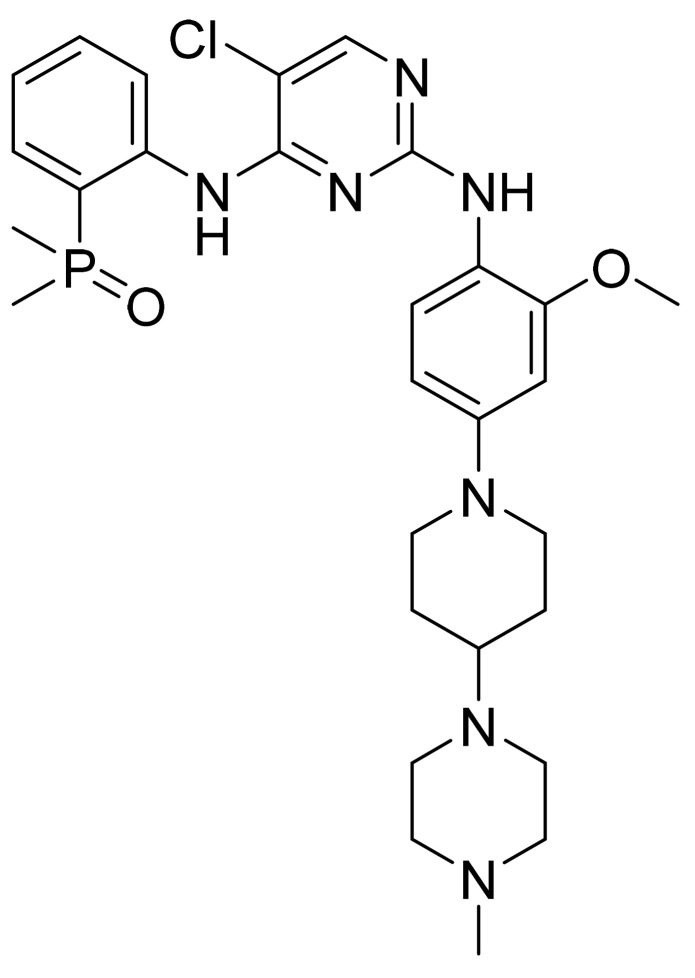
Brigatinib (5-chloro-*N*4-[2-(dimethylphosphoryl)phenyl]-*N*2-{2-methoxy-4[4-(4-methylpiperazin-1-yl)piperidin-1-yl]phenyl}pyrimidine-2,4-diamine).

Brigatinib (Figure 36) is a piperazine-piperidine based pyrimidine derivative (MF: C_29_H_39_ClN_7_O_2_P; MW: 584.10; CAS Number: 1197953-54-0) [119]. **US9012462B2** narrates phosphorous compounds as PTKIs and their use in treating cancers. It claims brigatinib and its salts [120]. **US10385078B2** claims a stable and non-hygroscopic anhydrous crystalline form A of brigatinib suitable for pharmaceutical formulation development [121].

### 4.36. Midostaurin

**Figure 37:**
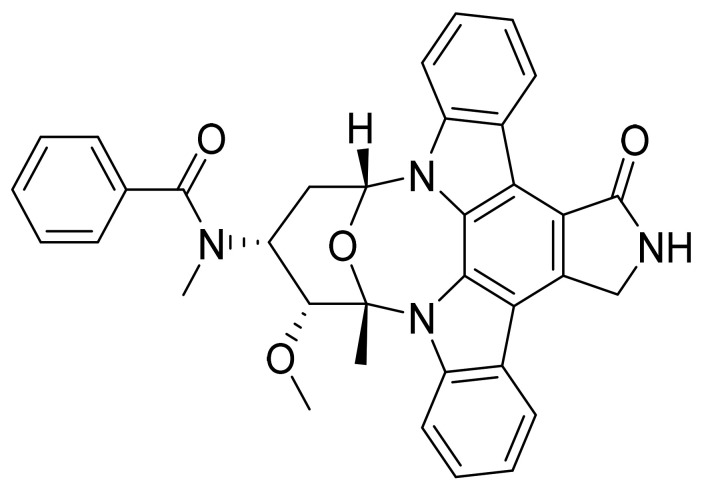
Midostaurin (*N*-[(2S,3R,4R,6R)-3-Methoxy-2-methyl-16-oxo-29-oxa-1,7,17-triazaoctacyclo[12.12.2.12,6.07,28.08,13.015,19.020,27.021,26]nonacosa-8,10,12,14,19,21,23,25,27-nonaen-4-yl]-*N*-methylbenzamide).

Midostaurin (Figure 37) is an indolocarbazole derivative (MF: C_35_H_30_N_4_O_4_; MW: 570.65; CAS Number: 120685-11-2) [122]. **US5093330A** relates to staurosporine derivatives, their salts, synthesis, and compositions encompassing them to treat cancer and inflammation. It discloses midostaurin [123]. **US7973031B2** claims a method for treating AML using a dosage form (a microemulsion, soft gel, or solid dispersion) of midostaurin, wherein the AML is characterized by deregulated FLT3 receptor tyrosine kinase activity [124].

### 4.37. Neratinib Maleate

**Figure 38:**
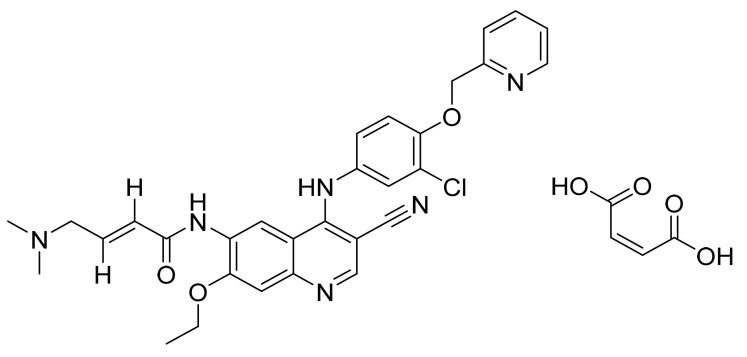
Neratinib maleate ((*E*)-*N*-{4-[3-chloro-4-(pyridin-2-ylmethoxy)anilino]-3-cyano-7-ethoxyquinolin-6-yl}-4-(dimethylamino)but-2-enamide maleate).

Neratinib maleate (Figure 38) is a pyridine based 4-aminoquinoline derivative (MF: C_30_H_29_ClN_6_O_3_·C_4_H_4_O_4_; MW: 673.11; CAS Number: 915942-22-2) [125]. **US7399865B2** reports substituted 3-cyanoquinoline compounds and their salts as inhibitors of HER-2 and EGFR to treat cancer. It claims neratinib and its salts [126].

### 4.38. Copanlisib Dihydrochloride

**Figure 39:**
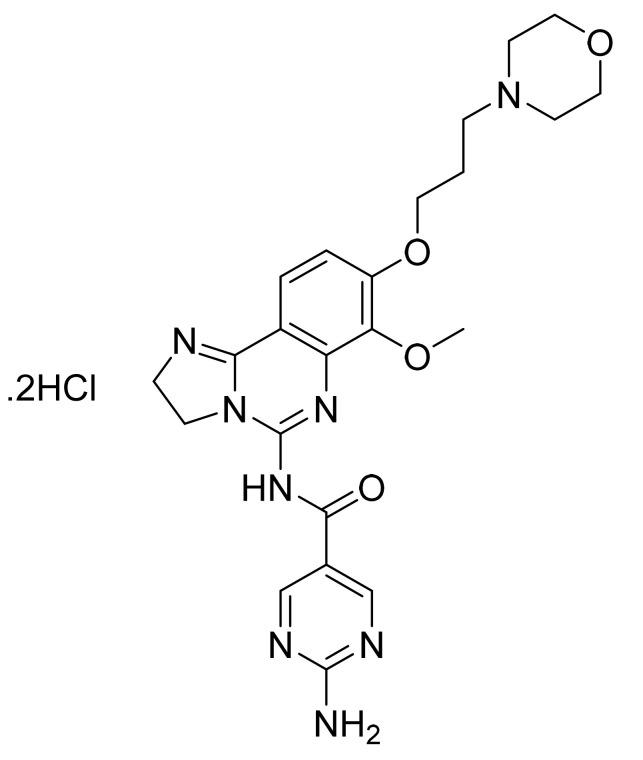
Copanlisib dihydrochloride (2-amino-*N*-{7-methoxy-8-[3-(morpholin-4-yl)propoxy]-2,3-dihydroimidazo[1,2-*c*]quinazolin-5-yl}pyrimidine-5-carboxamide dihydrochloride).

Copanlisib dihydrochloride (Figure 39) is a morpholine-pyrimidine based 2,3-dihydroimidazo[1,2-*c*]quinazoline derivative (MF: C_23_H_28_N_8_O_4_·2HCl; MW: 553.45; CAS Number: 1402152-13-9) [127]. **USRE46856E** (Reissue of US8466283B2) unveils 2,3-dihydroimidazo[1,2-*c*]quinazoline derivatives, pharmaceutical compositions comprising them, and the use of these compounds for treating hyperproliferative and angiogenesis disorders. It claims copanlisib and its salts [128]. **US10383876B2** claims copanlisib dihydrochloride salt that possesses technically advantageous properties (stability, solubility, hygroscopicity, etc.) to develop a quality pharmaceutical composition [129].

### 4.39. Abemaciclib

**Figure 40:**
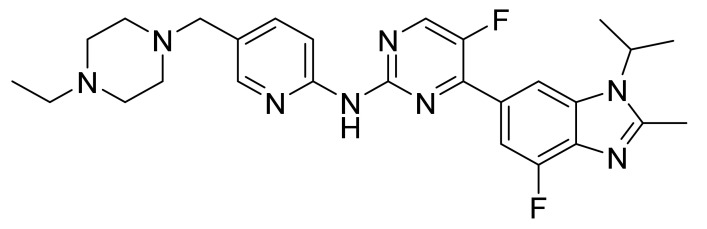
Abemaciclib (*N*-[5-[(4-ethyl-1-piperazinyl)methyl]-2-pyridinyl]-5-fluoro-4-[4-fluoro-2-methyl-1-(1-methylethyl)-1H-benzimidazol-6-yl]pyrimidin-2-amine).

Abemaciclib (Figure 40) is a piperazine-pyridine-pyrimidine based benzimidazole derivative (MF: C_27_H_32_F_2_N_8_; MW: 506.59; CAS Number: 1231929-97-7) [130]. **US7855211B2** reports piperazine-pyridine-pyrimidine based benzimidazole derivatives and salts thereof, a pharmaceutical formulation comprising them to treat cancers selected from the group colorectal cancer, breast cancer, NSCLC, prostate cancer, glioblastoma, MCL, CML, and AML. It claims abemaciclib and its salts [131].

### 4.40. Acalabrutinib

**Figure 41:**
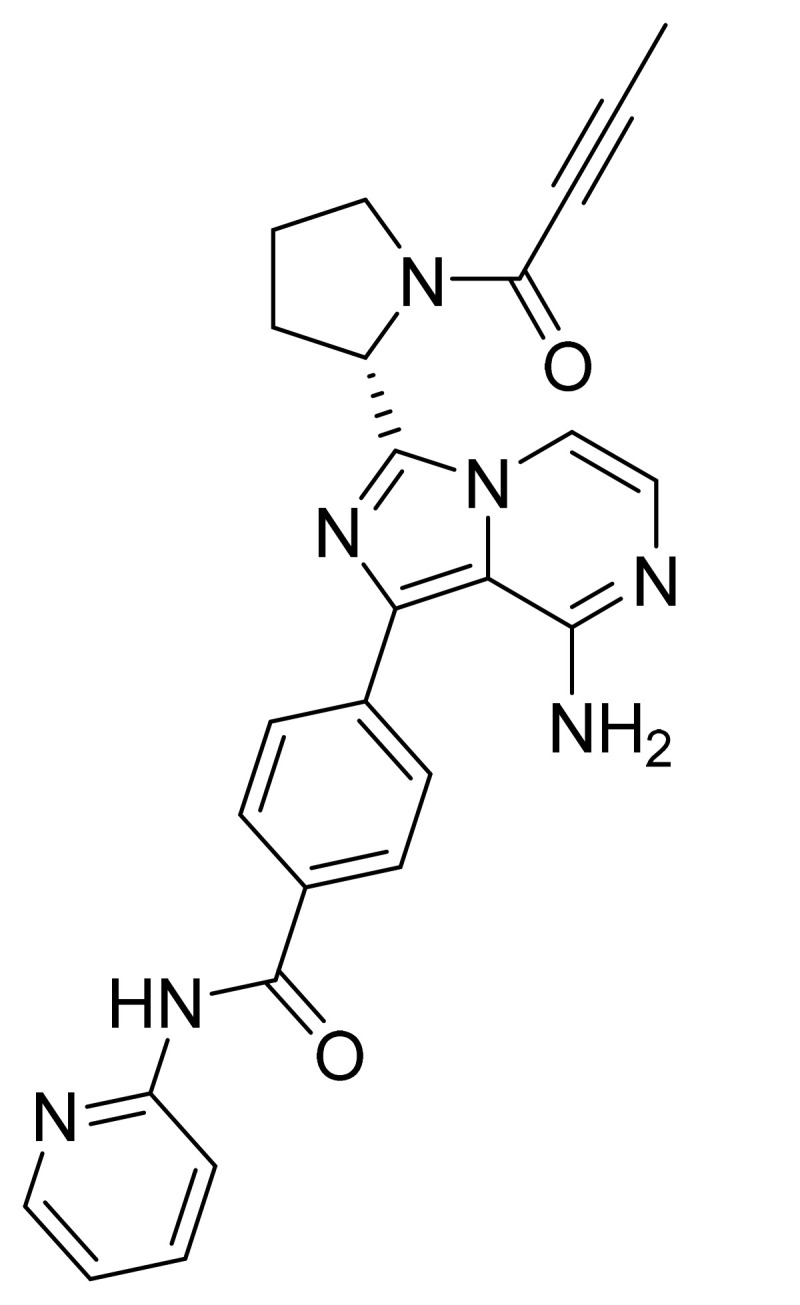
Acalabrutinib (4-{8-amino-3-[(2S)-1-(but-2-ynoyl)pyrrolidin-2-yl]imidazo[1,5-*a*]pyrazin-1-yl}-*N*-(pyridin-2-yl)benzamide).

Acalabrutinib (Figure 41) is a pyrrolidine-pyridine based imidazo[1,5-*a*]pyrazine derivative (MF: C_26_H_23_N_7_O_2_; MW: 465.51; CAS Number: 1420477-60-6) [132]. **US9290504B2** provides 4-imidazopyridazin-1-yl-benzamides for the treatment of BTK mediated disorders. It claims acalabrutinib and its salts [133]. **US9796721B2** claims a stable and non-hygroscopic anhydrate crystal form of acalabrutinib as having advantageous parameters for making quality pharmaceutical compositions [134].

### 4.41. Netarsudil Dimesylate

**Figure 42:**
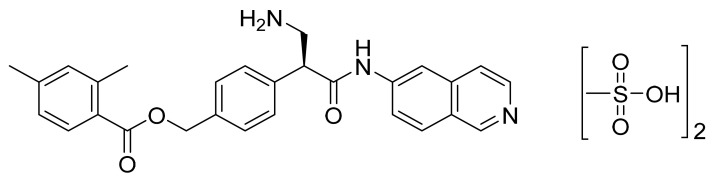
Netarsudil dimesylate ((S)-4-(3-amino-1-(isoquinolin-6-ylamino)-1-oxopropan-2-yl)benzyl-2,4-dimethylbenzoate dimesylate).

Netarsudil dimesylate (Figure 42) is an isoquinoline based beta-amino acid derivative (MF: C_30_H_35_N_3_O_9_S_2_; MW: 645.74; CAS Number: 1422144-42-0) [135]. **US8394826B2** relates to isoquinoline amide and benzamide based compounds as dual inhibitors of Rho kinase and a monoamine transporter (MAT), useful in treating diseases like glaucoma and cancer. It claims netarsudil [136]. **US9415043B2** claims a chemically stable and water-soluble dimesylate salt of netarsudil that can provide a quality ophthalmic solution [137].

### 4.42. Baricitinib

**Figure 43:**
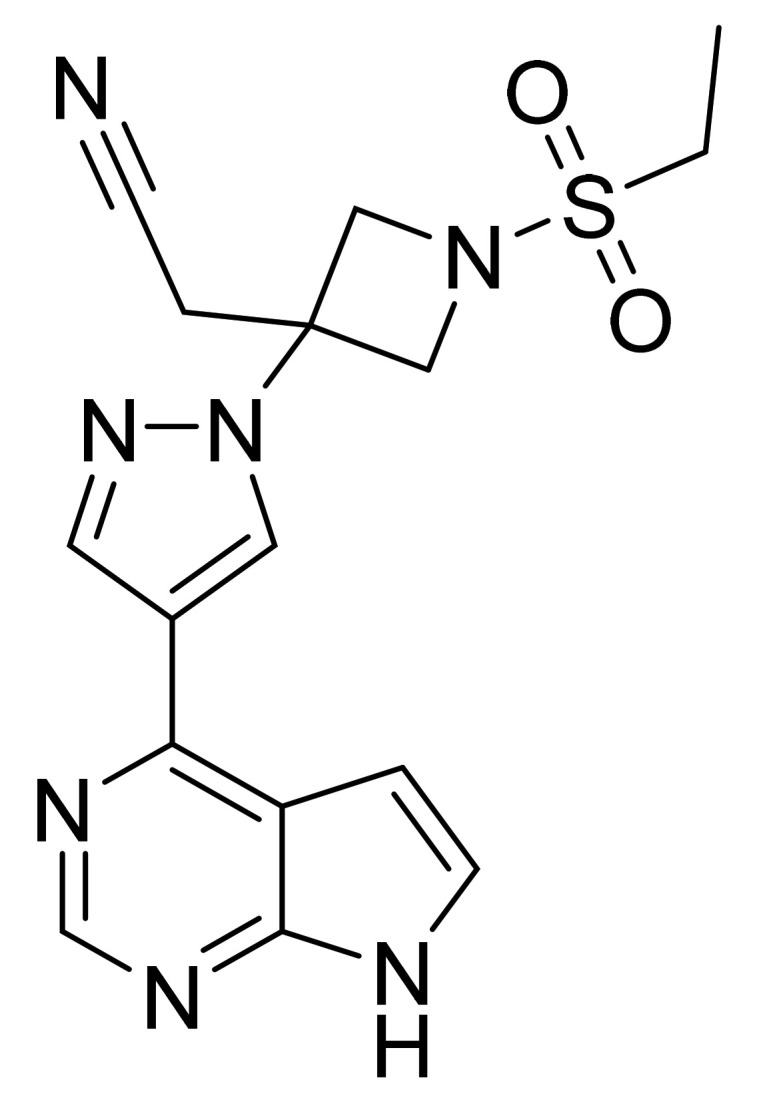
Baricitinib ({1-(ethylsulfonyl)-3-[4-(7H-pyrrolo[2,3-*d*]pyrimidin-4-yl)-1H-pyrazol-1-yl]azetidin-3-yl}acetonitrile).

Baricitinib (Figure 43) is a pyrazole-azetidine based pyrrolo[2,3-*d*]pyrimidine derivative (MF: C_16_H_17_N_7_O_2_S; MW: 371.42; CAS Number: 1187594-09-7) [138]. **US8158616B2** provides azetidine derivatives as JAK inhibitors, synthetic methods, and compositions encompassing them to treat inflammatory and autoimmune disorders, along with cancer. It claims baricitinib and its salts [139].

### 4.43. Binimetinib

**Figure 44:**
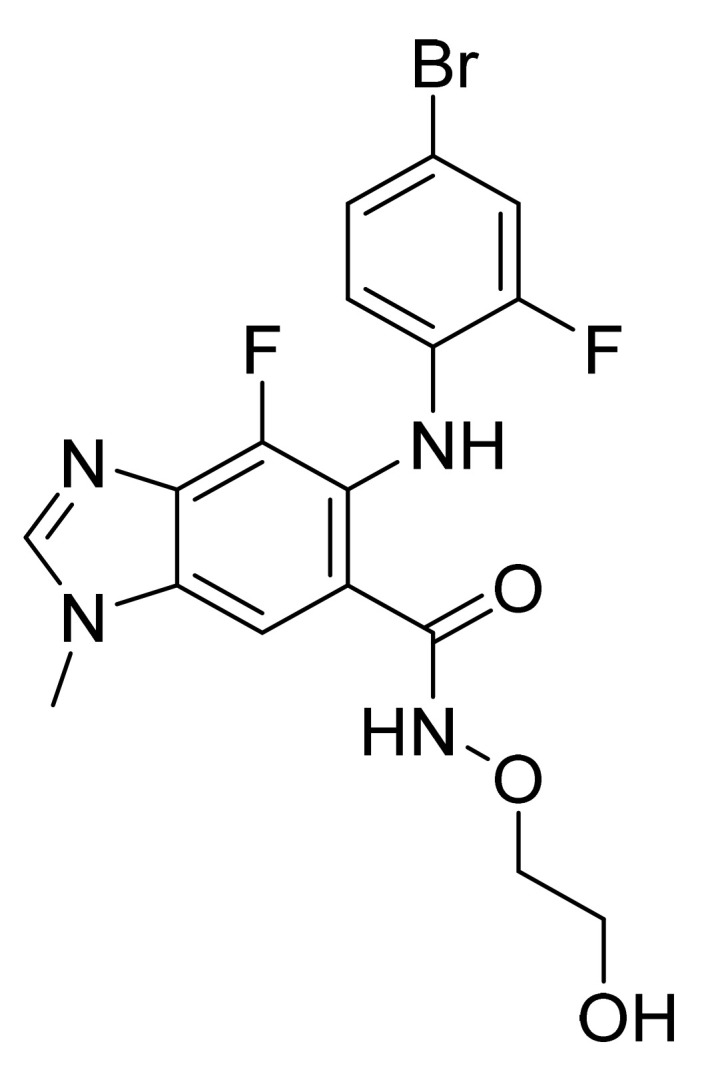
Binimetinib (5-[(4-bromo-2-fluorophenyl)amino]-4-fluoro-*N*-(2-hydroxyethoxy)-1-methyl-1H-benzimidazole-6-carboxamide).

Binimetinib (Figure 44) is a benzimidazole derivative (MF: C_17_H_15_BrF_2_N_4_O_3_; MW: 441.2; CAS Number: 606143-89-9) [140]. **US7777050B2** states alkylated (1H-Benzoimidazol-5-yl)-(4-substituted-phenyl)-amine derivatives, helpful in managing sicknesses like cancer. It claims binimetinib and pharmaceutically acceptable salts thereof [141]. **US9562016B2** claims a crystallized form of binimetinib with better purity and an enhanced physical characteristic, beneficial in pharmaceutical dosage form preparation [142].

### 4.44. Dacomitinib Monohydrate

**Figure 45:**
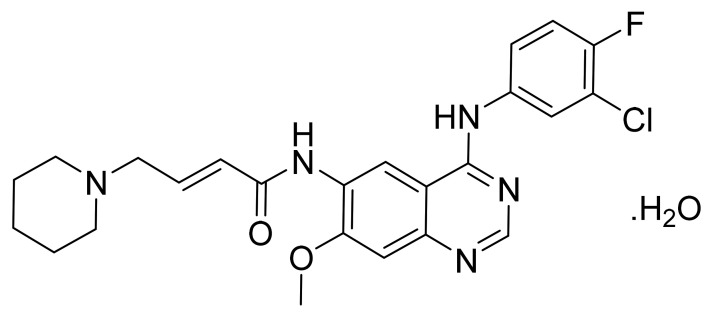
Dacomitinib monohydrate ((2*E*)-*N*-{4-[(3-Chloro-4-fluorophenyl)amino]-7-methoxyquinazolin-6-yl}-4-(piperidin-1-yl)but-2-enamide monohydrate).

Dacomitinib monohydrate (Figure 45) is a piperidine based quinazolinamine derivatives (MF: C_24_H_25_ClFN_5_O_2_·H_2_O; MW: 487.95; CAS Number: 1042385-75-0) [143]. **US7772243B2** unveils 4-anilino-6-substituted alkenoylamino-quinazoline compounds as TKIs to treat proliferative diseases, including cancer and restenosis endometriosis and psoriasis. It claims dacomitinib and its salts [144].

### 4.45. Encorafenib

**Figure 46:**
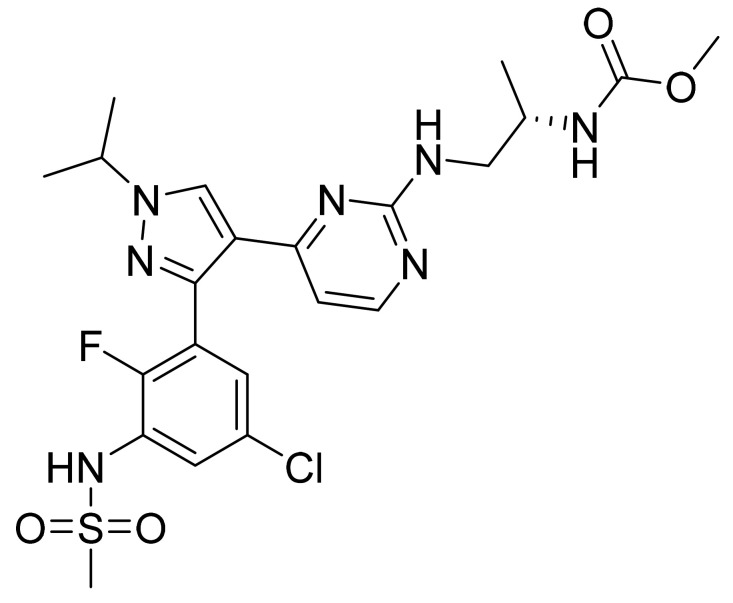
Encorafenib (*N*-{(2S)-1-[(4-{3-[5-chloro-2-fluoro-3-(methanesulfonamido)phenyl]-1-(propan-2-yl)-1H-pyrazol-4-yl}pyrimidin-2-yl)amino]propan-2-yl}carbamate).

Encorafenib (Figure 46) is a pyrazole based pyrimidine derivative (MF: C_22_H_27_ClFN_7_O_4_S; MW: 540; CAS Number: 1269440-17-6) [145]. **US8501758B2** provides pyrazole based pyrimidine and pharmaceutical compositions comprising them to treat disorders associated with the deregulated activity of B-Raf. It claims encorafenib and its salts [146].

### 4.46. Fostamatinib Disodium Hexahydrate

**Figure 47:**
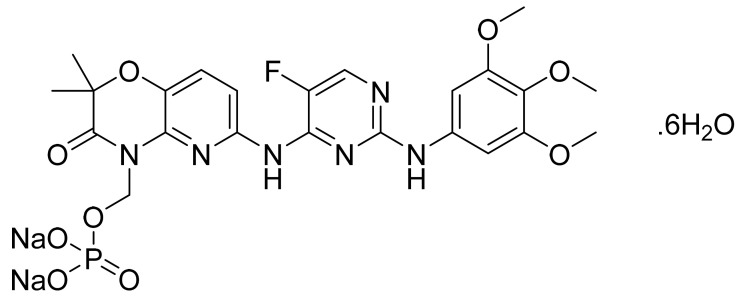
Fostamatinib disodium hexahydrate (Disodium (6-[[5-fluoro-2-(3,4,5-trimethoxyanilino) pyrimidin-4-yl]amino]-2,2-dimethyl-3-oxo-pyrido[3,2-*b*][1,4]oxazin-4-yl)methyl phosphate hexahydrate).

Fostamatinib disodium hexahydrate (Figure 47), a phosphate prodrug of tamatinib, is a pyrimidine based pyrido[3,2-*b*][1,4]oxazine derivative (MF: C_23_H_24_FN_6_Na_2_O_9_P·6H_2_O; MW: 732.52; CAS Number: 914295-16-2) [147]. **US7449458B2** reports prodrugs of pharmacologically active 2,4-pyrimidinediamine derivatives, intermediates thereof, the process of manufacturing them, and pharmaceutical compositions comprising them to treat diseases mediated by the activation of PTKs. It claims fostamatinib disodium hexahydrate, which has increased solubility concerning the parent phosphate prodrug [148]. **US8163902B2** claims a thermodynamically stable crystalline form of fostamatinib disodium hexahydrate that is stable over a wide range of relative humidity and requires substantial heating to lose its water molecules. This property makes it a suitable API to develop the desired dosage form [149].

### 4.47. Duvelisib Hydrate

**Figure 48:**
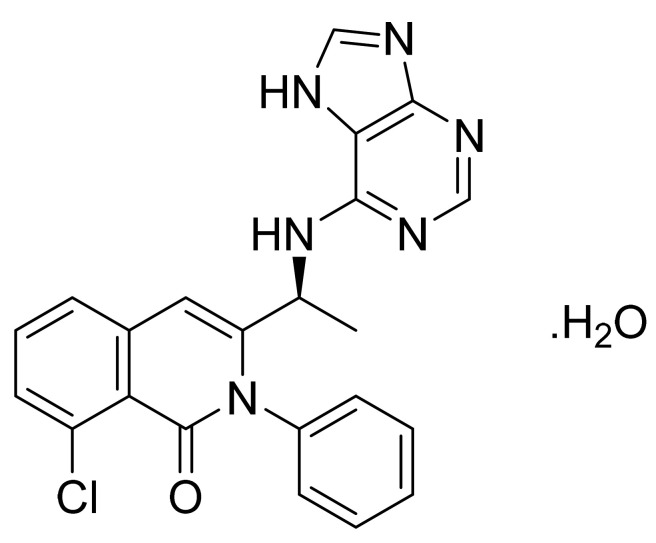
Duvelisib hydrate ((S)-3-(1-(9H-purin-6-ylamino)ethyl)-8-chloro-2-phenylisoquinolin-1(2H)-one hydrate).

Duvelisib hydrate (Figure 48) is a purine based isoquinolone derivative (MF: C_22_H_17_ClN_6_O·H_2_O; MW: 434.88; CAS Number: 1201438-56-3) [150]. **US8193182B2** provides isoquinolin-1(2H)-one derivatives as modulators of PI3 kinase activity and pharmaceutical compositions comprising them to treat diseases associated with P13 kinase activity. It claims duvelisib and its salts [151]. **USRE46621E** (Reissue of US8809349B2) claims physically and chemically stable polymorphs of duvelisib, salt, solvate, or hydrate that do not readily decompose or change in chemical makeup or physical state for more than 60 months and are suitable to develop the desired dosage forms of the API [152].

### 4.48. Gilteritinib Fumarate

**Figure 49:**
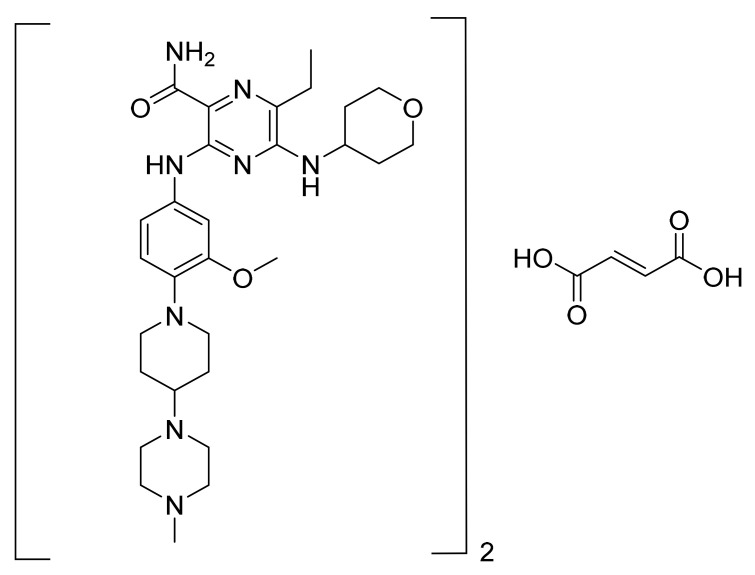
Gilteritinib fumarate (2-Pyrazinecarboxamide 6-ethyl-3-[[3-methoxy-4-[4-(4-methyl-1-piperazinyl)-1-piperidinyl] phenyl]amino]-5-[(tetrahydro-2H-pyran-4-yl)amino]-, (2*E*)-2-butenedioate (2:1)).

Gilteritinib fumarate (Figure 49) piperazine-piperidine based pyrazine carboxamide derivative (MF: (C_29_H_44_N_8_O_3_)_2_·C_4_H_4_O_4_; MW: 1221.50; CAS Number: 1254053-84-3) [153]. **US8969336B2** states diamino heterocyclic carboxamide derivatives as having outstanding inhibitory activity against EML4-ALK fusion proteins for use in cancer therapy. It claims gilteritinib and its salts [154]. The gilteritinib fumarate salt is stable in heat, humidity, and storage conditions.

### 4.49. Larotrectinib Sulfate

**Figure 50:**
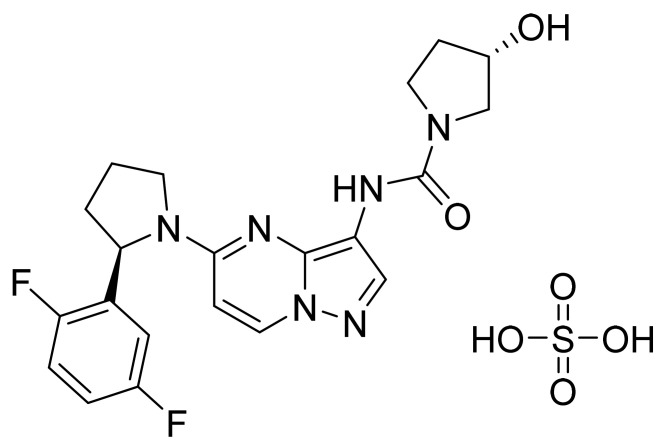
Larotrectinib sulfate ((3S)-*N*-{5-[(2R)-2-(2,5-difluorophenyl)-1-pyrrolidinyl]pyrazolo[1,5-*a*]pyrimidin-3-yl}-3-hydroxy-1-pyrrolidinecarboxamide sulfate).

Larotrectinib sulfate (Figure 50) is a pyrrolidine based pyrazolo[1,5-*a*]pyrimidine derivative (MF: C_21_H_24_F_2_N_6_O_6_S; MW: 526.51; CAS Number: 1223405-08-0) [155]. **US9127013B2** relates to pyrazolo[1,5-*a*] pyrimidine derivatives as TRK family PTKIs that are useful to treat cancer, inflammation, and certain infectious diseases. It claims larotrectinib sulfate specifically [156]. **US10172861B2** claims crystalline larotrectinib sulfate having stable physicochemical properties, which can be used to develop quality dosage forms [157].

### 4.50. Lorlatinib

**Figure 51:**
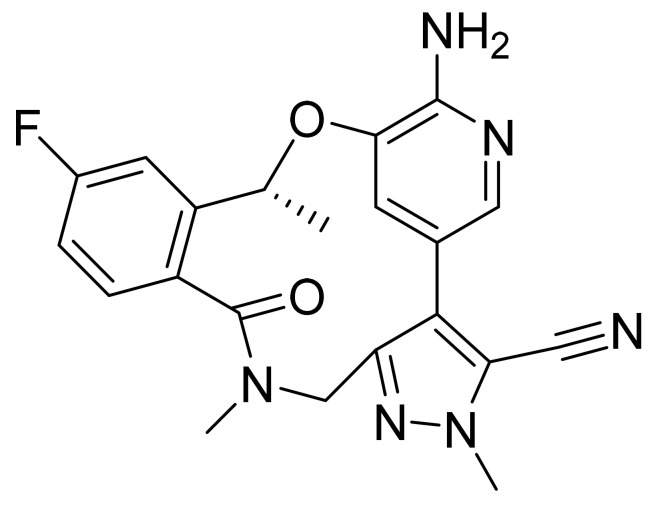
Lorlatinib ((10R)-7-amino-12-fluoro-2,10,16-trimethyl-15-oxo-10,15,16,17-tetrahydro-2H-4,8-methenopyrazolo[4,3-H][2,5,11]benzoxadiazacyclotetradecine-3-carbonitrile).

Lorlatinib (Figure 51) is a pyrazole-pyridine based benzoxadiazacyclotetradecine derivative (MF: C_21_H_19_FN_6_O_2_; MW: 406.41; CAS Number: 1223403-58-4) [158]. **US8680111B2** discloses macrocyclic compounds as inhibitors of ALK and/or EML4-ALK and their pharmaceutical composition to treat illnesses linked with the deregulation of ALK and EML4-ALK. It claims lorlatinib and its salts [159]. **US10420749B2** claims crystalline polymorphs of lorlatinib having high crystallinity and purity, low hygroscopicity, and favorable dissolution and mechanical properties to develop quality pharmaceutical formulations [160].

### 4.51. Entrectinib

**Figure 52:**
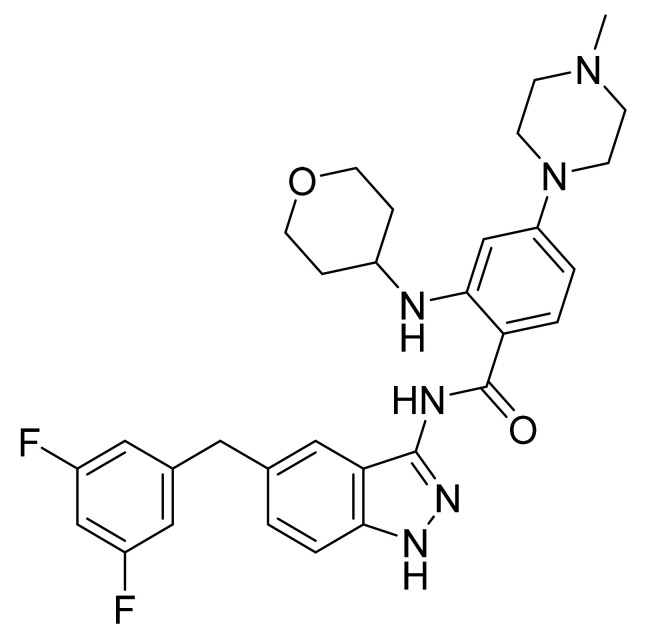
Entrectinib (*N*-[5-(3,5-difluorobenzyl)-1H-indazol-3-yl]-4-(4-methylpiperazin-1-yl)-2-(tetrahydro-2H-pyran-4-ylamino)benzamide).

Entrectinib (Figure 52) is a tetrahydropyran-piperazine based indazole derivative (MF: C_31_H_34_F_2_N_6_O_2_; MW: 560.64; CAS Number: 1108743-60-7) [161]. **US8299057B2** discloses indazole derivatives as potent PKIs that are useful in anticancer therapy. It claims entrectinib and its salts [162]. **US10738037B2** claims a crystalline Form 4 of entrectinib that exhibits greater thermodynamic stability at a temperature of about 40 °C than other known polymorphs and offers advantages in preparing dosage forms [163].

### 4.52. Upadacitinib Hemihydrate

**Figure 53:**
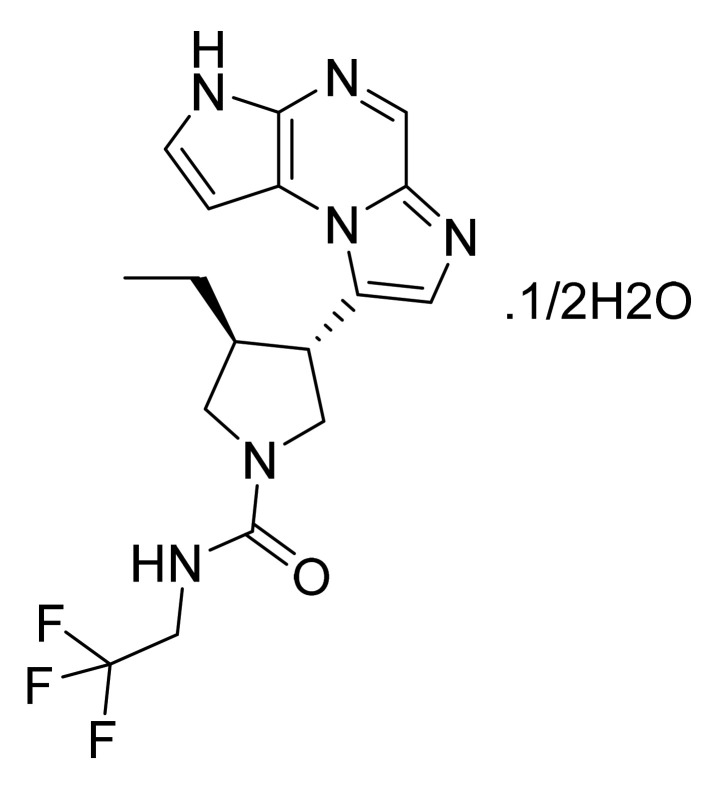
Upadacitinib hemihydrate ((3S,4R)-3-Ethyl-4-(3H-imidazo[1,2-*a*]pyrrolo[2,3-*e*]pyrazin-8-yl)-*N*-(2,2,2-trifluoroethyl)pyrrolidine-1-carboxamide hydrate (2:1)).

Upadacitinib hemihydrate (Figure 53) is an imidazo[1,2-*a*]pyrrolo[2,3-*e*]pyrazine based pyrrolidine derivative (MF: C_17_H_19_F_3_N_6_O·½H_2_O; MW: 389.38; CAS Number: 1310726-60-3) [164]. **USRE47221E** (Reissue of US8426411B2) describes tricyclic compounds that inhibit JAK family kinase activity for treating diseases, including rheumatoid arthritis, multiple sclerosis, and psoriasis. It claims upadacitinib [165]. **US9951080B2** claims physicochemically stable crystalline hemihydrate of upadacitinib having solid-state properties to develop quality pharmaceutical dosage forms [166].

### 4.53. Alpelisib

**Figure 54:**
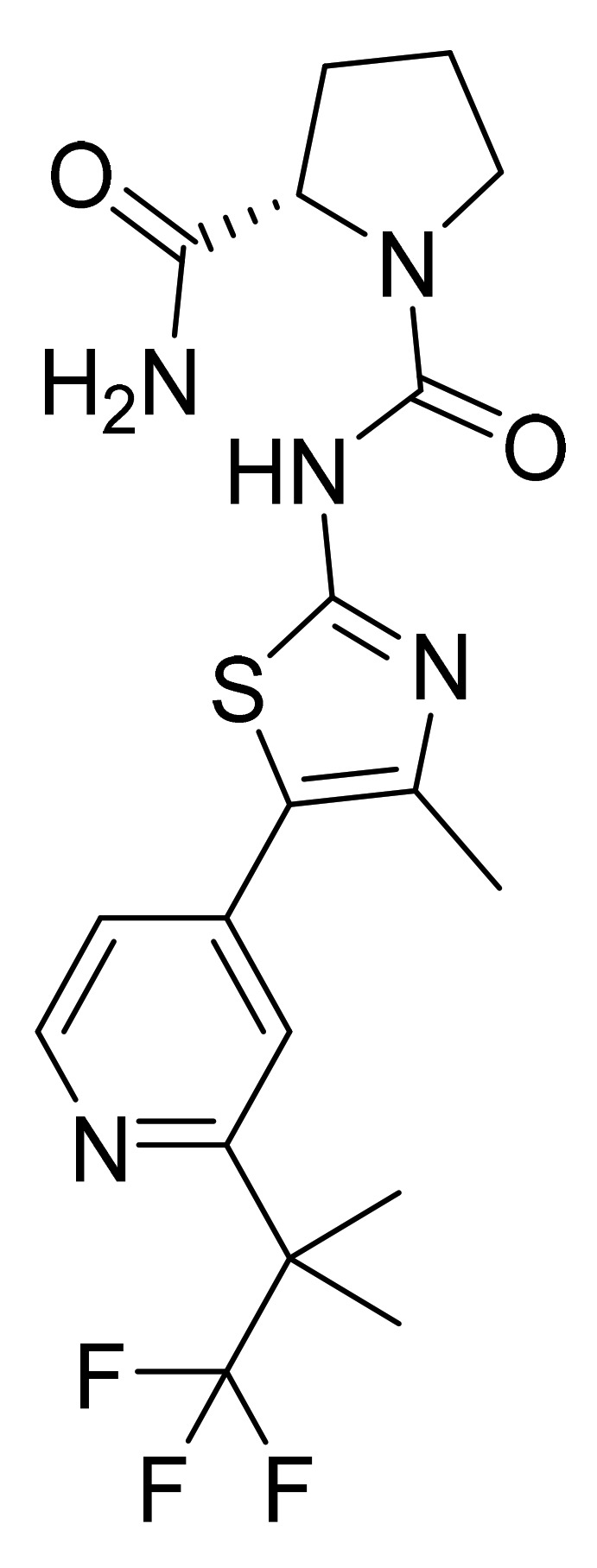
Alpelisib ((2S)-*N*1-[4-Methyl-5-[2-(2,2,2-trifluoro-1,1-dimethylethyl)-4-pyridinyl]-2-thiazolyl]-1,2-pyrrolidine dicarboxamide).

Alpelisib (Figure 54) is a pyridine-thiazole based pyrrolidine derivative (MF: C_19_H_22_F_3_N_5_O_2_S; MW: 441.47; CAS Number: 1217486-61-7) [167]. **US8227462B2** unveils pyrrolidine-1,2-dicarboxamide derivatives for the treatment of illnesses ameliorated by inhibition of PI3Ks. It claims alpelisib in a free form and its salts [168].

### 4.54. Erdafitinib

**Figure 55:**
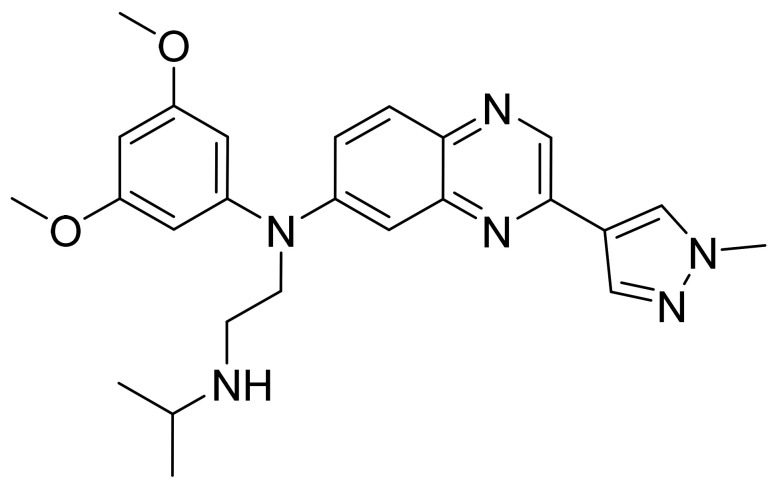
Erdafitinib (*N*-(3,5-dimethoxyphenyl)-3-(1-methyl-1H-pyrazol-4-yl)-*N*-{2-[(propan-2-yl)amino]ethyl}quinoxalin-6-amine).

Erdafitinib (Figure 55) is a pyrazole based quinoxaline derivative (MF: C_25_H_30_N_6_O_2_; MW: 446.56; CAS Number: 1346242-81-6) [169]. **US8895601B2** relates to pyrazole based quinoxaline derivatives and their pharmaceutical compositions to treat diseases like cancer. It claims erdafitinib and its salts [170].

### 4.55. Pexidartinib Hydrochloride

**Figure 56:**
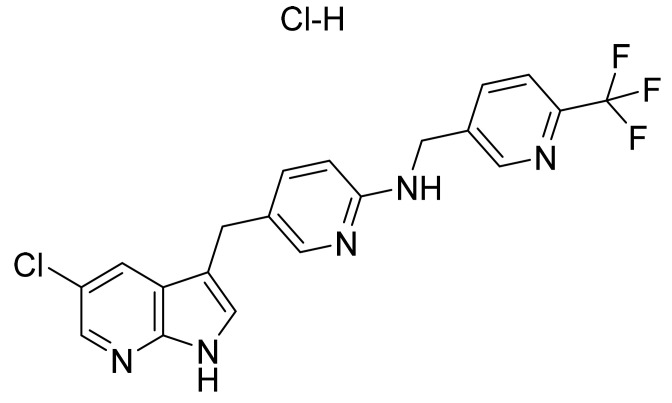
Pexidartinib hydrochloride (5-[(5-Chloro-1H-pyrrolo[2,3-*b*]pyridin-3-yl)methyl]-*N*-{[6-(trifluoromethyl)pyridin-3-yl]methyl}pyridin-2-amine monohydrochloride).

Pexidartinib hydrochloride (Figure 56) is a pyrrolo[2,3-*b*]pyridine based pyridine derivative (MF: C_20_H_15_ClF_3_N_5_·HCl; MW: 454.28; CAS Number: 1029044-16-3) [171]. **US9169250B2** provides fused azacyclic compounds as dual inhibitors of c-FMS and c-KIT to treat diseases that arise due to deregulation of c-FMS and c-KIT. It claims pexidartinib hydrochloride [172]. **US9802932B2** claims a stable crystalline form of pexidartinib hydrochloride having attributes for developing a quality pharmaceutical composition [173].

### 4.56. Fedratinib Dihydrochloride Monohydrate

**Figure 57:**
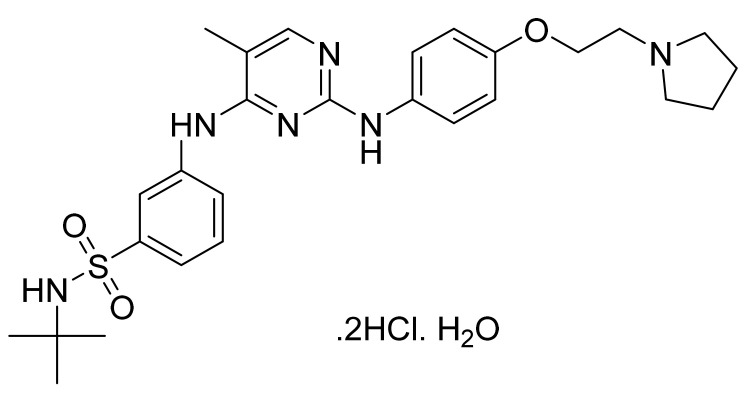
Fedratinib dihydrochloride monohydrate (*N*-tert-butyl-3-{[5-methyl-2-({4-[2-(pyrrolidin-1-yl)ethoxy]phenyl}amino)pyrimidin-4-yl]amino}benzene-1-sulfonamide dihydrochloride monohydrate).

Fedratinib dihydrochloride monohydrate (Figure 57) is a pyrrolidine-pyrimidine based benzenesulfonamide derivative (MF: C_27_H_36_N_6_O_3_S·2HCl·H_2_O; MW: 615.62; CAS Number: 1374744-69-0) [174]. **US7528143B2** unveils biaryl m-pyrimidine compounds as an inhibitor of the JAK family and their pharmaceutical compositions to treat diseases mediated by modulation of JAK activity. It claims fedratinib and its salts [175].

### 4.57. Zanubrutinib

**Figure 58:**
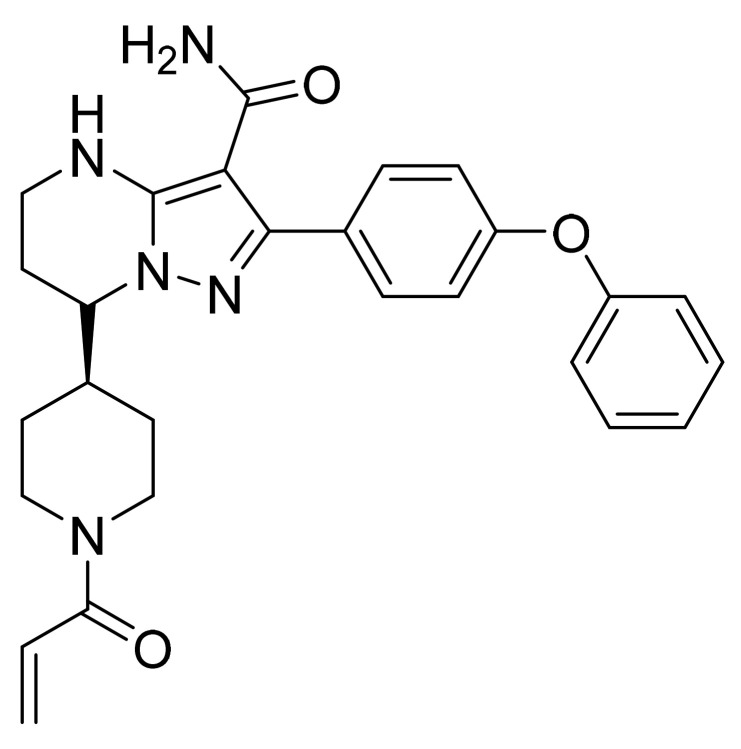
Zanubrutinib ((S)-7-(1-Acryloylpiperidin-4-yl)-2-(4-phenoxyphenyl)-4,5,6,7-tetrahydropyrazolo[1,5-*a*]pyrimidine-3-carboxamide).

Zanubrutinib (Figure 58) is a piperidine based pyrazolo[1,5-*a*]pyrimidine derivative (MF: C_27_H_29_N_5_O_3_; MW: 471.56; CAS Number: 1691249-45-2) [176]. **US9447106B2** states substituted pyrazolo[1,5-*a*]pyrimidines as BTK modulators and used these compounds to treat diseases intervened by BTK. It claims zanubrutinibas and its salts [177].

### 4.58. Avapritinib

**Figure 59:**
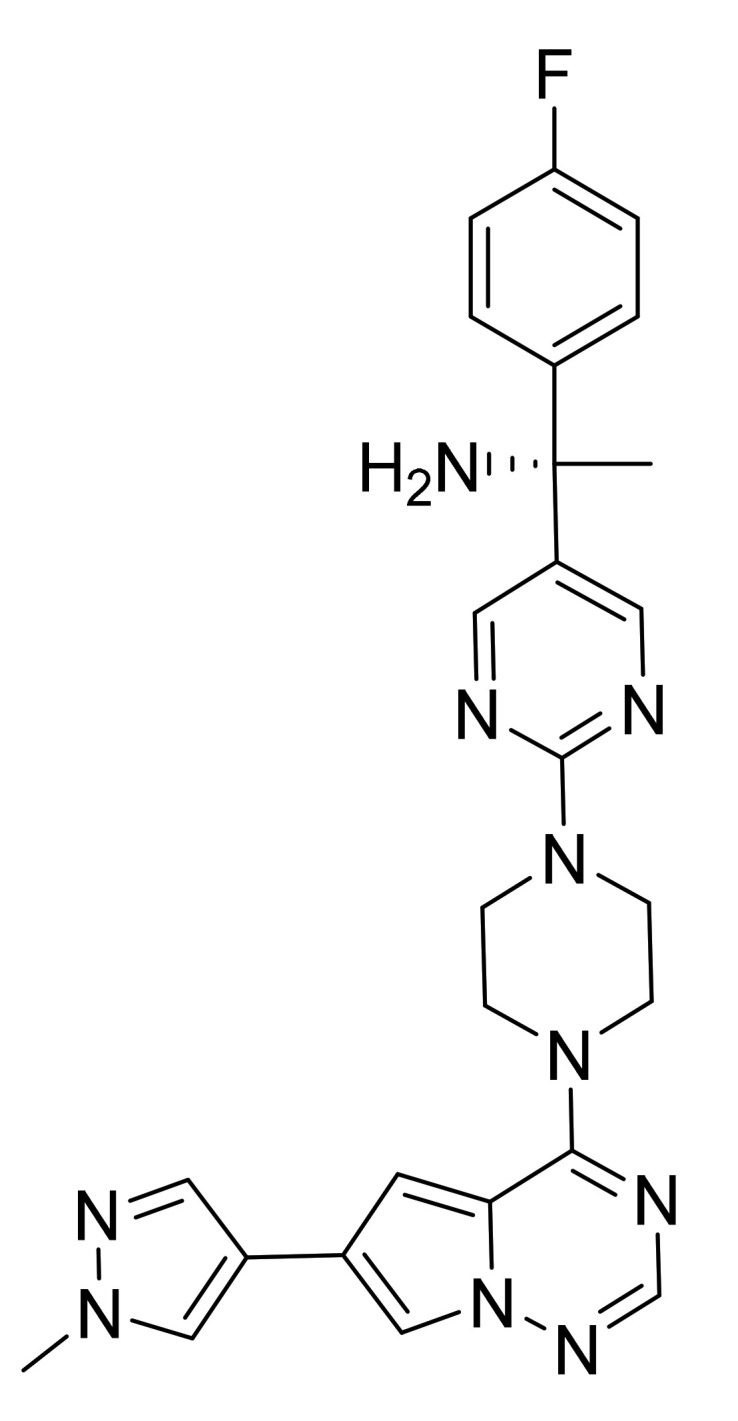
Avapritinib ((S)-1-(4-fluorophenyl)-1-(2-(4-(6-(1-methyl-1H-pyrazol-4-yl)pyrrolo[2,1-*f*][1,2,4]triazin-4-yl)piperazin-yl)pyrimidin-5-yl)ethan-1-amine).

Avapritinib (Figure 59) is a pyrazole-piperazine-pyrimidine based pyrrolo[2,1-*f*][1,2,4]triazine derivative (MF: C_26_H_27_FN_10_; MW: 498.57; CAS Number: 1703793-34-3) [178]. **US9944651B2** refers to piperazine-based pyrrolo[2,1-*f*][1,2,4]triazine derivatives for treating conditions like mastocytosis and mast cell diseases by modifying the activity of KIT. It claims avapritinib and its salts [179].

### 4.59. Selumetinib Sulfate

**Figure 60:**
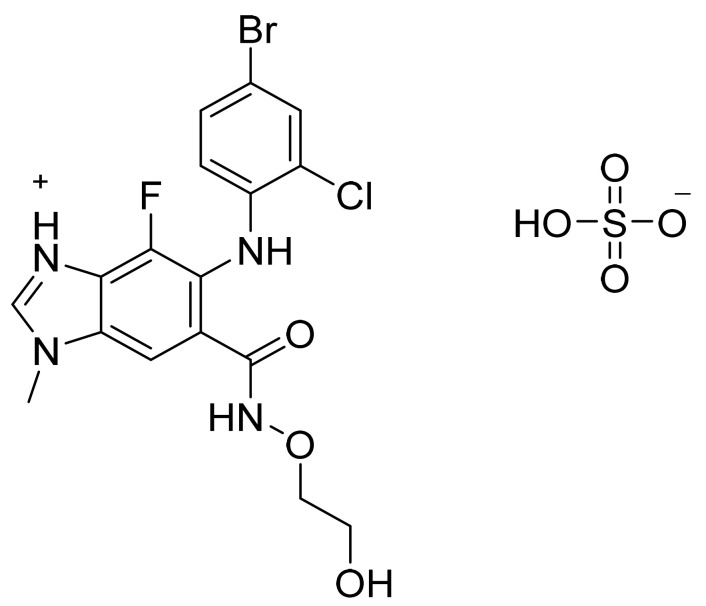
Selumetinib sulfate (5-[(4-bromo-2-chlorophenyl)amino]-4-fluoro-6-[(2-hydroxyethoxy)carbamoyl]-1-methyl-1H-benzimidazol-3-ium hydrogen sulfate).

Selumetinib sulfate (Figure 60) is a benzimidazole derivative (MF: C_17_H_17_BrClFN_4_O_7_S; MW: 555.76; CAS Number: 943332-08-9) [180]. **US7425637B2** reports *N*3-alkylated benzimidazole compounds that inhibit MEK and are helpful to treat cancer and inflammation. It claims selumetinib and its salts [181]. **US9156795B2** claims a stable crystalline hydrogen sulfate salt of selumetinib with enhanced solubility and bioavailability, making it a suitable API to develop desired pharmaceutical dosage forms [182].

### 4.60. Pemigatinib

**Figure 61:**
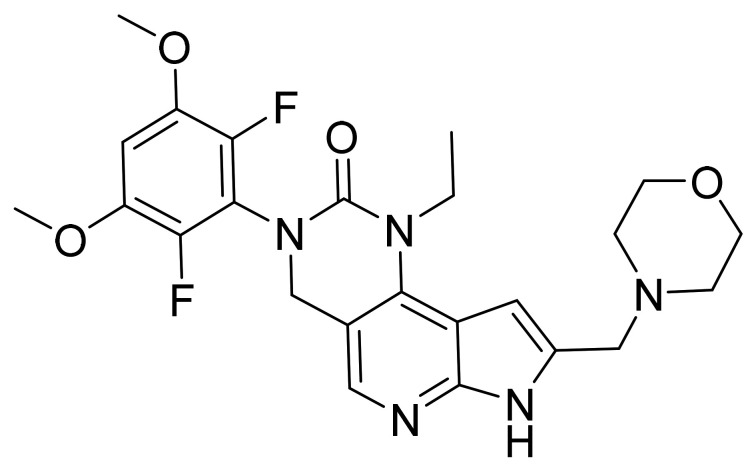
Pemigatinib (3-(2,6-difluoro-3,5-dimethoxyphenyl)1-ethyl-8-(morpholin-4-ylmethyl)-1,3,4,7-tetrahydro-2H-pyrrolo[3′,2′:5,6]pyrido[4,3-*d*]pyrimidin-2-one).

Pemigatinib (Figure 61) is a morpholine based pyrrolo[3′,2′:5,6]pyrido[4,3-*d*]pyrimidine derivative (MF: C_24_H_27_F_2_N_5_O_4_; MW: 487.5; CAS Number: 1513857-77-6) [183]. **US9611267B2** relates to tricyclic compounds as inhibitors of FGFR, useful in ailments facilitated by FGFR malfunctioning like cancer. It claims pemigatinib and its salts [184].

### 4.61. Tucatinib

**Figure 62:**
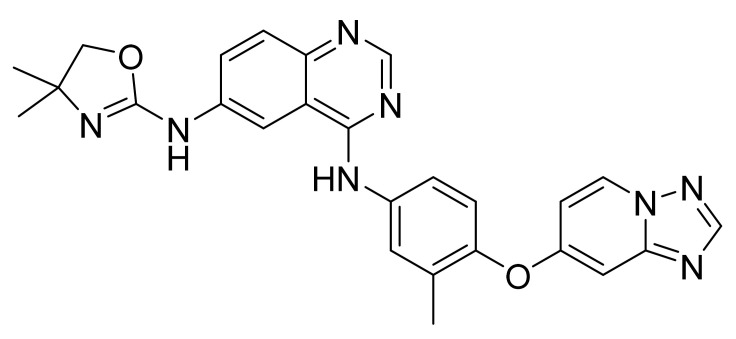
Tucatinib (*N*6-(4,4-dimethyl-4,5-dihydro-1,3-oxazol-2-yl)-*N*4-(3-methyl-4-{[1,2,4]triazolo[1,5-*a*]pyridin-7-yloxy}phenyl)quinazoline-4,6-diamine).

Tucatinib (Figure 62) is a quinazoline-oxazoline based triazolo[1,5-*a*]pyridine derivative (MF: C_26_H_24_N_8_O_2_; MW: 480.52; CAS Number: 937263-43-9) [185]. **US8648087B2** discloses *N*4-phenyl-quinazoline-4-amine derivatives as TKIs to treat cancer and inflammation. It claims tucatinib [186].

### 4.62. Capmatinib Dihydrochloride Monohydrate

**Figure 63:**
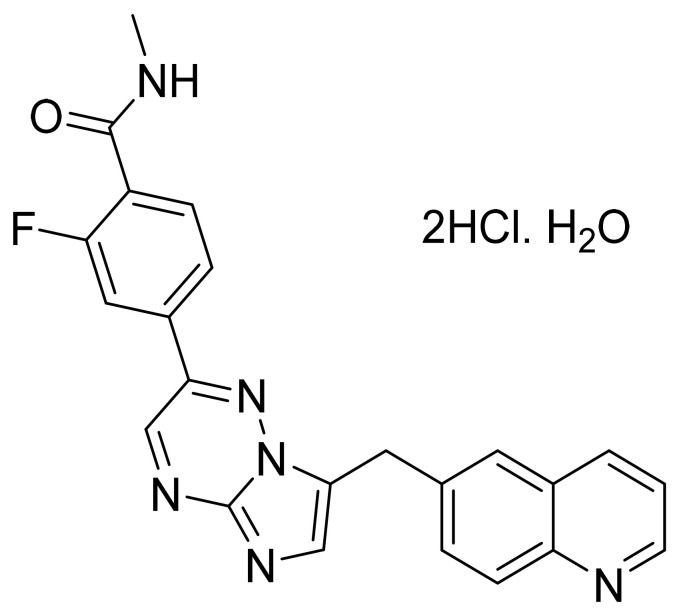
Capmatinib dihydrochloride monohydrate (2-fluoro-*N*-methyl-4-{7-[(quinolin-6-yl)methyl]imidazo[1,2-*b*][1,2,4]triazin-2-yl}benzamide dihydrochloride monohydrate).

Capmatinib dihydrochloride monohydrate (Figure 63) is an imidazo[1,2-*b*][1,2,4]triazine based quinoline derivative (MF: C_23_H_21_Cl_2_FN_6_O_2_; MW: 503.36; CAS Number: 1865733-40-9) [187]. **US7767675B2** reveals imidazotriazines and imidazopyrimidines as MET inhibitors and their pharmaceutical compositions useful in cancer treatment. It claims capmatinib and its salts [188]. **US8420645B2** claims a stable capmatinib dihydrochloride monohydrate with pharmaceutical attributes to manufacture quality pharmaceutical formulations [189].

### 4.63. Selpercatinib

**Figure 64:**
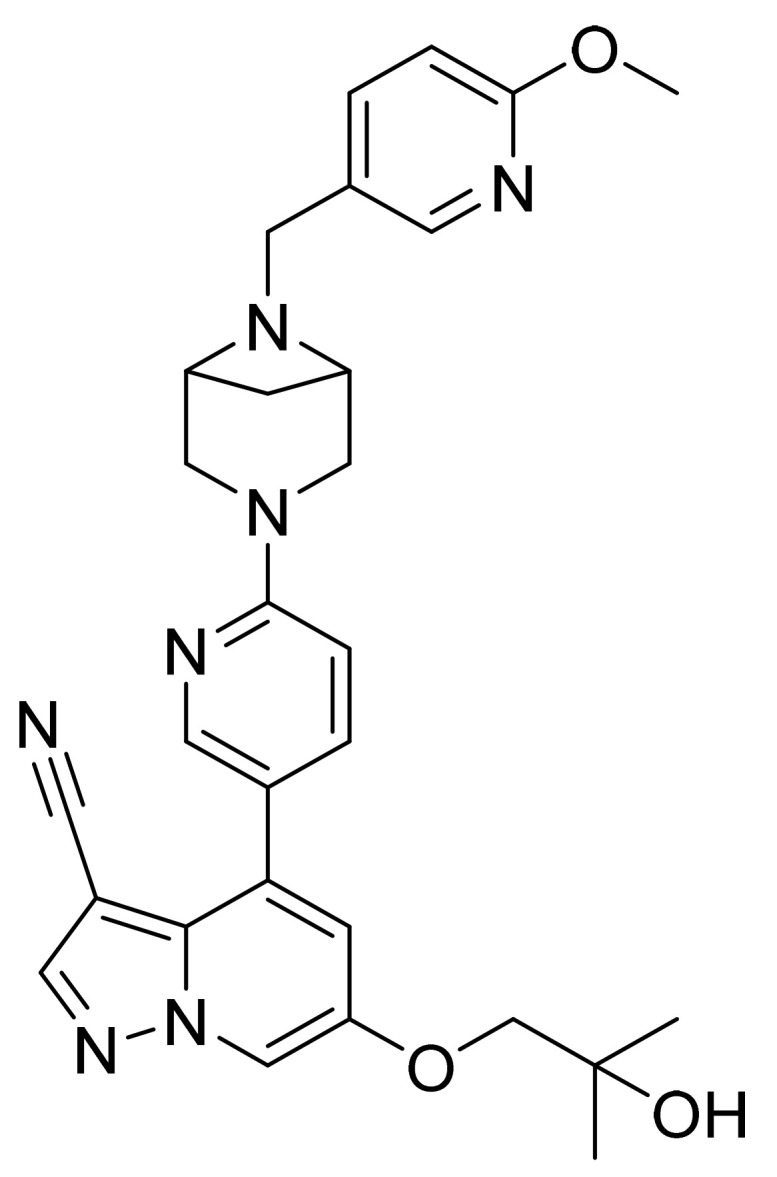
Selpercatinib (6-(2-hydroxy-2-methylpropoxy)-4-(6-(6-((6-methoxypyridin-3-yl)methyl)-3,6-diazabicyclo[3.1.1]heptan-3-yl)pyridin-3-yl)pyrazolo[1,5-*a*]pyridine-3-carbonitrile).

Selpercatinib (Figure 64) is a pyridine-diazabicycloheptane based pyrazolo[1,5-*a*]pyridine derivative (MF: C_29_H_31_N_7_O_3_; MW: 525.61; CAS Number: 2152628-33-4) [190]. **US10112942B2** uncovers pyrazolo[1,5-*a*]pyridines as RET inhibitors, useful to treat RET-associated diseases. It claims selpercatinib and its salts [191]. **US10584124B2** claims a stable crystalline polymorph of selpercatinib that is useful for developing pharmaceutical formulations [192].

### 4.64. Ripretinib

**Figure 65:**
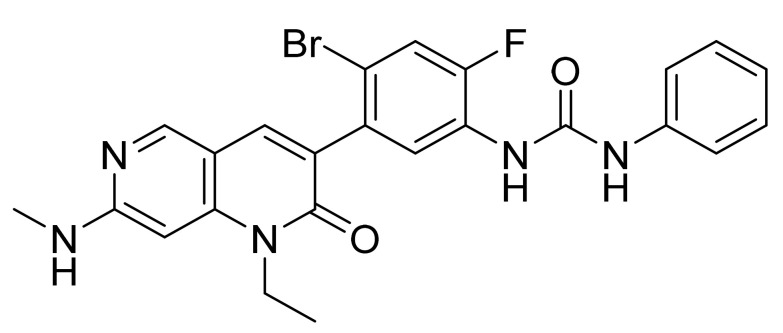
Ripretinib (1-(4-bromo-5-[1-ethyl-7-(methylamino)-2-oxo-1,2-dihydro-1,6-naphthyridin-3-yl]-2-fluorophenyl)-3-phenylurea).

Ripretinib (Figure 65) is a naphthyridine based phenylurea derivative (MF: C_24_H_21_BrFN_5_O_2_; MW: 510.36; CAS Number: 1442472-39-0) [193]. **US8461179B1** uncovers dihydronaphthyridine derivatives that inhibit c-KIT and that have utility to treat GIST, mast cell leukemia, or mastocytosis. It claims ripretinib and its salts [194].

### 4.65. Pralsetinib

**Figure 66:**
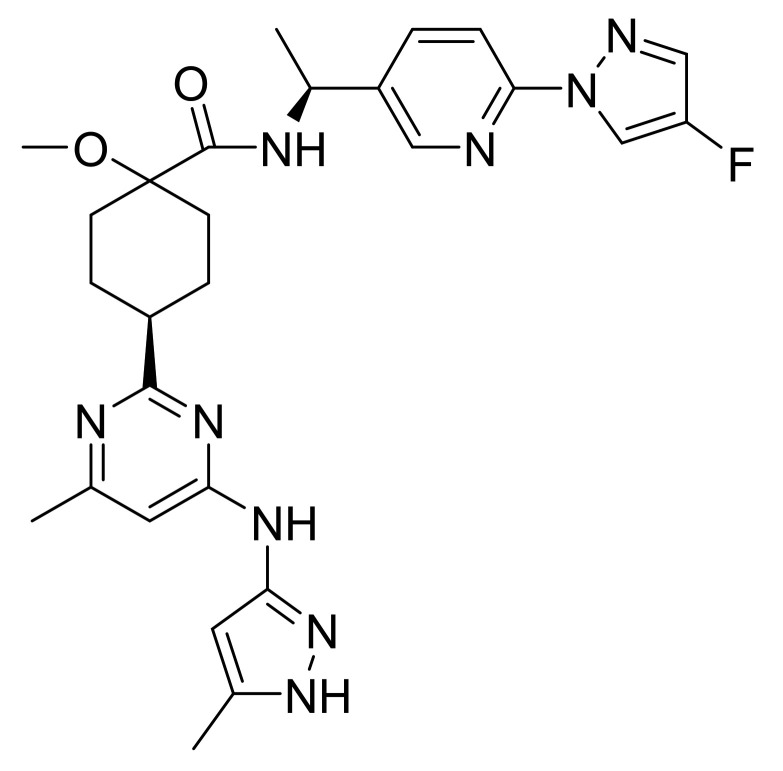
Pralsetinib ((Cis)-*N*-((S)-1-(6-(4-fluoro-1H-pyrazol-1-yl)pyridin-3-yl)ethyl)-1-methoxy-4-(4-methyl-6-(5-methyl-1H-pyrazol-3-ylamino)pyrimidin-2-yl)cyclohexane carboxamide).

Pralsetinib (Figure 66) is a pyridine-pyrimidine based pyrazole derivative (MF: C_27_H_32_FN_9_O_2_; MW: 533.61; CAS Number: 2097132-94-8) [195]. **US10030005B2** discloses pyrazole based RET inhibitors and their pharmaceutical compositions to treat a condition mediated by aberrant RET activity, e.g., cancer. It claims pralsetinib [196].

### 4.66. Trilaciclib Dihydrochloride

**Figure 67:**
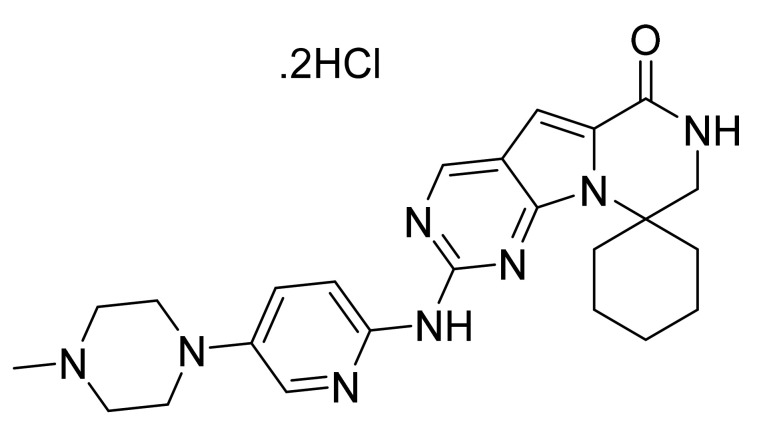
Trilaciclib dihydrochloride (2′-{[5-(4-methylpiperazin-1-yl)pyridin-2-yl]amino}-7′,8′-dihydro-6′H-spiro[cyclohexane-1,9′-pyrazino[1′,2′:1,5]pyrrolo[2,3-*d*]pyrimidin]-6′-one).

Trilaciclib dihydrochloride (Figure 67) is a piperazine-pyridine based pyrazino[1′,2′:1,5]pyrrole derivative (MF: C_24_H_30_N_8_O·2HCl; MW: 519.48; CAS Number: 1977495-97-8) [197]. **US8598186B2** reveals tricyclic compounds as CDK inhibitors, which have utility in the treatment of disorders intervened by CDK malfunction like cancer. It claims trilaciclib and its salts [198].

### 4.67. Tepotinib Hydrochloride Monohydrate

**Figure 68:**
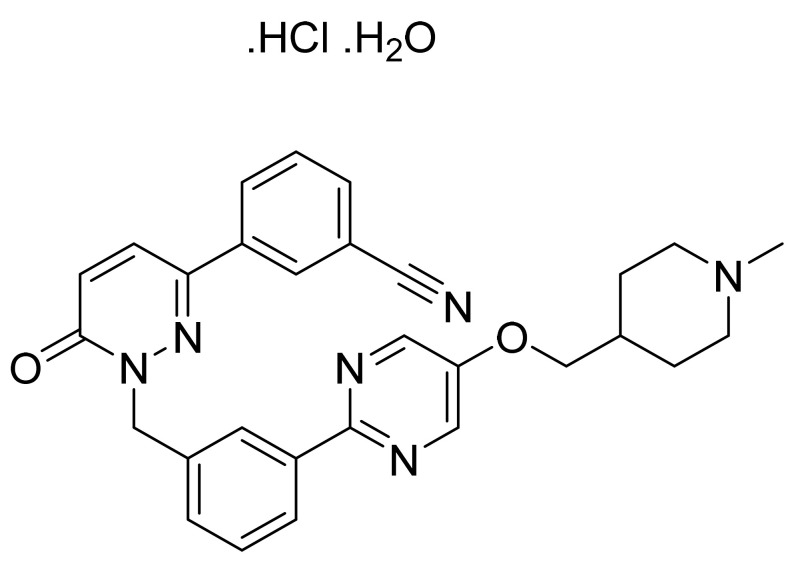
Tepotinib hydrochloride monohydrate (3-{1-[(3-{5-[(1-methylpiperidin-4-yl)methoxy]pyrimidin-2-yl}phenyl)methyl]-6-oxo-1,6-dihydropyridazin-3-yl}benzonitrile hydrochloride monohydrate).

Tepotinib hydrochloride monohydrate (Figure 68) is a piperidine-pyrimidine based dihydropyridazine derivative (MF: C_29_H_28_N_6_O_2_·HCl·H_2_O; MW: 547.05; CAS Number: 1946826-82-9) [199]. **US8580781B2** reveals certain pyridazinones as MET inhibitors to treat tumors. It claims tepotinibor and its salts [200]. Tepotinib hydrochloride hydrate is claimed explicitly in **US8329692B2** [201].

### 4.68. Umbralisib Tosylate

**Figure 69:**
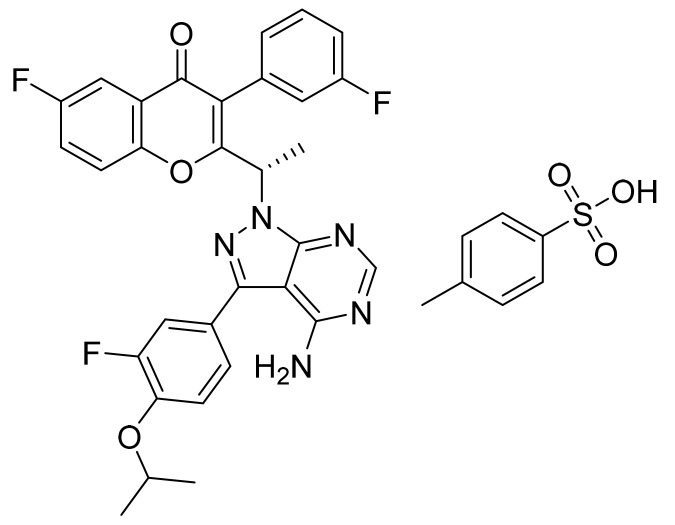
Umbralisib tosylate ((S)-2-(1-(4-amino-3-(3-fluoro-4-isopropoxyphenyl)-1H-pyrazolo[3,4-*d*]pyrimidin-1-yl)-ethyl)-6-fluoro-3-(3-fluorophenyl)-4H-chromen-4-one tosylate)).

Umbralisibtosylate (Figure 69) is a chromen-4-one based pyrazolo[3,4-*d*]pyrimidine derivative (MF: C_38_H_32_F_3_N_5_O_6_S; 743.75; 1532533-72-4) [202]. **US10570142B2** provides pyrazolo[3,4-*d*]pyrimidines as inhibitors of PI3K_δ_ and their pharmaceutical compositions to treat PI3K_δ_ mediated disorders. It claims umbralisib tosylate having at least 95% enantiomeric excess [203]. **US10414773B2** unveils a stable crystalline form of umbralisib tosylate possessing specified particle sizes with enhanced solubility and improved pharmacokinetics. This property makes it suitable to prepare a quality oral dosage form [204].

### 4.69. Tivozanib Hydrochloride Monohydrate

**Figure 70:**
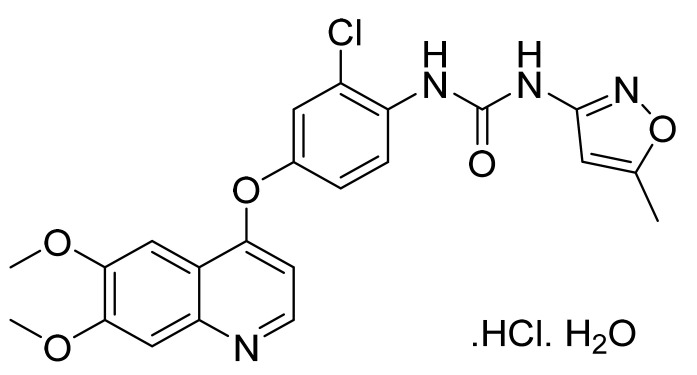
Tivozanib hydrochloride monohydrate (1-{2-chloro-4-[(6,7-dimethoxyquinolin-4-yl)oxy]phenyl}-3-(5-methylisoxazol-3-yl)urea hydrochloride monohydrate).

Tivozanib hydrochloride monohydrate (Figure 70) is an isoxazole base quinoline derivative (MF: C_22_H_19_ClN_4_O_5_·HCl·H_2_O; MW: 509.34; CAS Number: 682745-41-1) [205]. **US6821987B2** and **US7211587B2** unveil quinoline derivatives having azolyl group, useful for treating tumors, chronic rheumatism, psoriasis, and Kaposi’s sarcoma. These patents claim tivozanib and its salts [206,207]. **US7166722B2** claims a physically stable crystalline form of tivozanib hydrochloride monohydrate stable under high temperature and humidity. This form is suitable for developing quality dosage forms [208].

### 4.70. Infigratinib Phosphate

**Figure 71:**
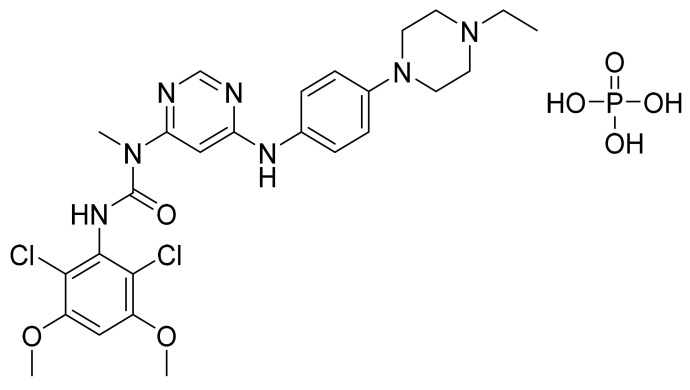
Infigratinib phosphate (3-(2,6-dichloro-3,5-dimethoxyphenyl)-1-{6-[4-(4-ethylpiperazin-1-yl)phenylamino]pyrimidin-4-yl}-1-methylurea phosphate).

Infigratinib (Figure 71) is a piperazine based pyrimidine derivative (MF: C_26_H_31_Cl_2_N_7_O_3_. H_3_PO_4_; MW: 658.47; CAS Number: 1310746-10-1) [209]. **US8552002B2** claims infigratinib and its salts [210]. **US9067896B2** claims a monophosphoric acid salt of infigratinib as well as its anhydrous crystalline polymorph (Form A) and amorphous polymorph. The stability and physicochemical parameters of the crystalline Form A were better than other disclosed polymorphs [211].

## 5. Expert Opinion

In 2001, USFDA approved the marketing of the first clinical PKI, imatinib. From 2001 to 31 May 2021, about 70 PKIs have been approved by the USFDA (Table 2). The USFDA has also approved antibodies as PKIs such as trastuzumab and bevacizumab. A few antibodies are also in the clinical trial (amivantamab and patritumab). This review is limited to small molecules as PKIs. Accordingly, USFDA approved antibodies such as PKIs have not been discussed here. The physicochemical properties of about 55 USFDA approved PKIs from 2001 to 2020 have been described in the literature [22,23]. However, these reports are silent about the patent data of the PKIs reported therein. 

According to the patent literature, and the data presented in Table 2 and Table 3, the major players that developed the marketed PKIs include Novartis (imatinib, lapatinib, everolimus, nilotinib, pazopanib, trametinib, dabrafenib, ceritinib, ribociclib, midostaurin, alpelisib, capmatinib, and infigratinib), Pfizer (tofacitinib, palbociclib, dacomitinib, and lorlatinib), Astrazeneca (gefitinib, osimertinib, acalabrutinib, and selumetinib), Bayers (sorafenib, regorafenib, copanlisib, and larotrectinib), and PF Prism (temsirolimus, crizotinib, axitinib, and bosutinib). Nearly 535 PKs have been reported [6]. However, the major primary target of the approved PKIs includes ALK, BCR-Abel, B-RAF, BTK, CDK, EGFR, JAK, MEK, PDGFR, PI3K, RET, and VEGFR (Table 2). Accordingly, there remains a large number of unexplored PKs. Some KIs have specificity for multiple kinases and are called multikinase inhibitors (MKIs), such as sunitinib, regorafenib, imatinib, sorafenib, axitinib, lenvatinib, cabozantinib, vandetanib, and pazopanib. The MKIs are supposed to reduce the chances of developing resistance. However, they are also linked to causing adverse effects in patients, for example, hypertension, gastric upset, and dermatological reactions [212]. The development of the covalent PKIs (ibrutinib, dacomitinib, osimertinib, afatinib, and neratinib) had been an unwilling strategy because they can bind to certain proteins and cause toxicity. Furthermore, the allosteric PKIs (trametinib, ascinimib, and selumetinib) are considered better than covalent inhibitors as they are not supposed to bind with other proteins. However, many new kinases have been identified possessing cysteine residues at their active sites. Therefore, the design of potent and selective covalent inhibitors may be useful against such kinases [213,214]. The pharmaceutical industries are trying to develop more potent and safer PKIs that can be used to treat many more PKs associated disorders with fewer adverse events [23]. Some example of PKIs, which are under development and/or waiting for the USFDA approval, include abrocitinib, belumosudil, dovitinib, sitravatinib, abivertinib, enzastaurin, rivoceranib (apatinib), asciminib, ensartinib, mobocertinib, momelotinib, pacritinib, quizartinib, vorolanib, GLPG3970, CA-4948, BAY1834845, BAY1830839, and PF-06650833 [213,214].

The PKIs contain one or more heterocyclic moieties in their structure that can explain the difference in their binding to the target and thus the spectrum of activity. The primary heterocyclic moieties include quinazoline, quinoline, isoquinoline, pyridine, pyrimidine, pyrazole, benzimidazole, indazole, imidazole, indole, carbazole, or their fused structures. This observation suggests that many clinical PKIs have been developed by the chemical modification of a formerly approved drug, and PKs are promiscuous targets. Further, most of the PKIs are marketed as acid-addition salts (hydrochloride, mesylate, tosylate, phosphate, malate, citrate, esylate, fumarate, succinate, and sulfate). This observation indicates the basic nature of the chemical nucleus of the PKIs.

The majority of the PKIs are approved to treat cancer and inflammatory disorders. Some of the PKIs have shown efficacy towards autoimmune diseases, Alzheimer’s disease (neflamapimod, tideglusib, and saracitinib), and Parkinson’s disease (DNL201). It is also expected that PKIs of PKC/WNK that control the activity of ion transporters may be developed to treat hypertension [214].

The malignant cells have genomic instability, which may cause the development of resistance to PKIs. This phenomenon is the reason for developing 2nd, 3rd, and later generations of PKIs targeting the equivalent PKs and their related disorders [212]. To combat resistance development, scientists are exploring different chemical templates and pharmacophores to develop novel PKIs [22]. Besides, inflammatory conditions do not exhibit genomic instability. Therefore, the PKIs, which are approved to treat inflammatory disorders, seldom demonstrate the development of resistance [22,23].

The main marketed dosage form of about 66 USFDA approved PKIs is either a tablet or capsule (Table 2). These are solid dosage forms. The quality of the formulation of a solid dosage form depends upon the solid-state properties (stability, solubility, compressibility, etc.) of the drug [215]. Therefore, many patents related to salts and polymorphs (mostly crystalline forms) of the USFDA approved PKIs have been obtained by the innovator companies. The innovator companies have done this to capture the market for a longer time.

The development of the PKIs is considered a medical breakthrough. However, the prices of these therapeutics cause financial toxicity. The financial burden can make the patients non-compliant with the treatment instructions as they may take lower doses than the prescribed doses. This causes failure of the treatment [216,217]. One way to avoid financial toxicity is to develop the generic version of a drug [218]. Currently, seven PKIs have been genericized (imatinib, erlotinib, sorafenib, dasatinib, lapatinib, temsirolimus, and everolimus) (Table 3). These generic versions must have lower prices than the innovator products. The data given in Table 3 also suggest that twelve more PKIs (gefitinib, sunitinib, pazopanib, vandetanib, axitinib, bosutinib, tofacitinib, idelalisib, nintedanib, lenvatinib, midostaurin, and neratinib) may be genericized by 2025 due to basic/compound/governing patent expiry or expiry of the drug exclusivity. It means by the end of 2025, 19 PKIs will have their generic version in the USA market. Besides, it is also expected that the generic version of about 48 PKIs will be available in the USA market by the end of 2030. Thus, it is hoped that the generic availability of these PKIs will reduce the financial toxicity on a patient.

**Figure 72:**
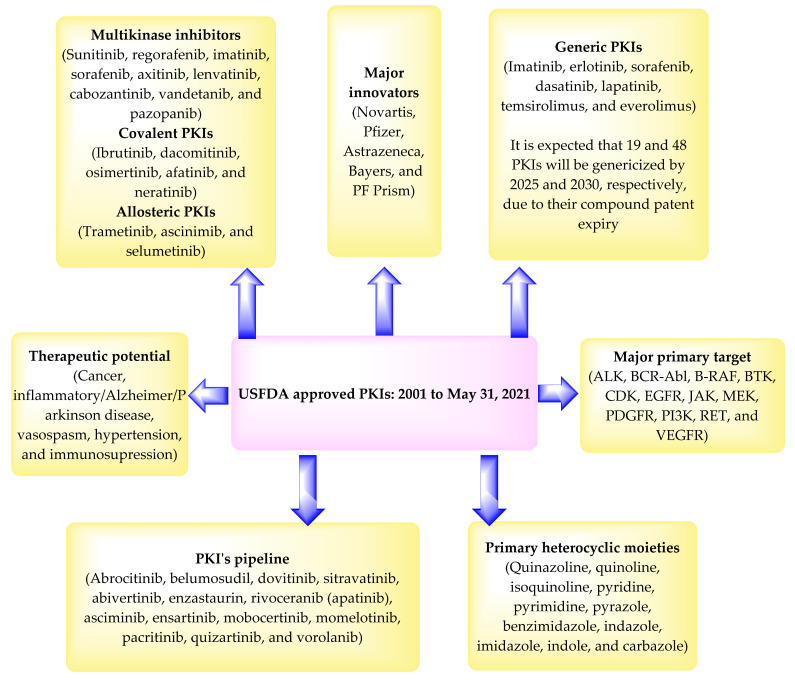
Summary of the USFDA approved PKIs.

Although great strides have been made in developing small molecule such as PKIs during the past 20 years, this field is still in its infancy. PKs are ubiquitous, and hence specificity has always been an issue regarding the design of new therapies targeting them. The major disadvantage of the existing PKIs is that they target a minor portion of the kinome, with countless clinically significant kinases missing validated inhibitors [22,23]. There are essential kinases without any inhibitors, and this is a critical area for further research. As the field advances during the next 20 years, one can anticipate that PKIs with many scaffolds, chemotypes, and pharmacophores will be developed. Other innovative strategies are also expected soon. A summary of the PKIs is provided in Figure 72.

In conclusion, there is a huge scope for discovering PKIs, and it will dominate other cancer discovery strategies for decades. The rate of discovery of better and selective PKIs having less propensity for resistance development will be faster than the last two decades because of the better understanding of the molecular and structural aspects of the human kinases. The development of PKIs to treat hypertension, Alzheimer’s disease, and Parkinson’s disease are foreseeable.

## Figures and Tables

**Table 2:** The Orange Book data of the USFDA approved PKIs.

Marketed Active Ingredient (Proprietary Name, Applicant)	Approved Dosage Form (Strength)	Approval Date (Marketing Status)	Primary Target ^#^	Approved Indication
Imatinib mesylate (Gleevec, Novartis Pharmaceuticals)	Tablet (100 and 400 mg of imatinib free base)	18 April 2003 (Prescription)	BCR-Abl	Many cancer types, including CML, Ph^+^-ALL, CEL, and GISTs
	Capsule (50 and 100 mg of imatinib free base)	10 May 2001 (Discontinued)		
Gefitinib (Iressa, AstraZeneca Pharmaceuticals)	Tablet (250 mg)	13 July 2015 (Prescription)	EGFR	NSCLC
	Tablet (250 mg)	5 May 2003 (Discontinued)		
Erlotinib hydrochloride (Tarceva, OSI Pharmaceuticals)	Tablet (25, 100, and 150 mg of erlotinib free base)	18 November 2004 (Prescription)	EGFR	Metastatic NSCLC and pancreatic cancer
Sorafenib tosylate (Nexavar, Bayer Healthcare Pharmaceuticals)	Tablet (200 mg of sorafenib free base)	20 December 2005 (Prescription)	VEGFR/BRAF	HCC, RCC, and DTC
Sunitinib malate (Sutent, CP Pharmaceuticals International)	Capsule (12.5, 25, 37.5, and 50 mg of sunitinib free base)	26 January 2006 (Prescription, 12.5, 25, and 50 mg) 31 March 2009 (Prescription, 37.5 mg)	VEGFR/PDGFR	GIST, RCC, and pNET
Dasatinib (Sprycel, Bristol Myers Squibb)	Tablet (20, 50, 70, 80, 100, and 140 mg)	28 June 2006 (Prescription)	BCR-Abl/ABL2	Ph^+^-CML
Lapatinib ditosylate (Tykerb, Novartis Pharmaceuticals)	Tablet (250 mg of lapatinib free base)	13 March 2007 (Prescription)	HER-1/HER-2/EGFR	Breast cancer
Temsirolimus (Torisel, PF Prism CV)	IV Solution (25 mg/mL)	30 May 2007 (Prescription)	FKBP12/mTOR	ARCC
Everolimus (Afinitor, Zortress, AfinitorDisperz, Novartis Pharmaceutical)	Tablet (2.5 mg, 5 mg, 7.5 mg, and 10 mg)	30 March 2009 (5 mg and 10 mg) 9 July 2010 (2.5 mg) 30 March 2012 (5 mg) (All are prescription products)	FKBP12/mTOR	pNET and RCC
	Tablet (0.25 mg, 0.5 mg, 0,75 mg, and 1 mg)	20 April 2010 (0.25, 0.5, and 0.75 mg) 10 August 2018 (1 mg) (All are prescription products)		
	Tablet for suspension (2 mg, 3 mg, and 5 mg)	29 August 2012 (Prescription)		
Nilotinib hydrochloride (Tasigna, Novartis Pharmaceuticals)	Capsule (50, 150, and 200 mg of nilotinib base)	29 October 2007 (200 mg Tablet) 17 June 2010 (150 mg Tablet) 22 March 2018 (50 mg Tablet) (All are prescription products)	BCR-Abl	Ph^+^-CML
Pazopanib hydrochloride (Votrient, Novartis Pharmaceuticals)	Tablet (200, and 400 mg of pazopanib base)	19 October 2009 (200 mg tablet, Prescription) (400 mg tablet has been discontinued)	VEGFR/PDGFR	RCC and STS
Vandetanib (Caprelsa, Genzyme Corp)	Tablet (100 mg and 300 mg)	6 April 2011 (Prescription)	VEGFR/EGFR	MTC
Vemurafenib (Zelboraf, Hoffmann La Roche)	Tablet (240 mg)	17 August 2011 (Prescription)	B-Raf	Melanoma with BRAF V600E mutation
Crizotinib (Xalkori, PF Prism CV)	Capsule (200 mg and 250 mg)	26 August 2011 (Prescription)	ALK/HGFR	NSCLC
Ruxolitinib phosphate (Jakafi, Incyte Corp)	Tablet (5 mg, 10 mg, 15 mg, 20 mg, and 25 mg of ruxolitinib free base)	16 November 2011 (Prescription)	JAK1/2/3 and Tyk2	Myelofibrosis and polycythemia vera
Axitinib (Inlyta, PF Prism CV)	Tablet (1 mg and 5 mg)	27 January 2012 (Prescription)	VEGFR/PDGFR	RCC
Bosutinib monohydrate (Bosulif, PF Prism CV)	Tablet (100 mg, 400 mg, and 500 mg of bosutinib free base)	4 September 2012 (100 and 500 mg) 27 October 2017 (400 mg) (All are prescription products)	BCR-Abl	Ph^+^-CML
Regorafenib (Stivarga, Bayer Healthcare Pharmaceuticals)	Tablet (40 mg)	27 September 2012 (Prescription)	VEGFR/TIE	Colorectal cancer, GIST, HCC, RCC and STS
Tofacitinib citrate (Xeljanz, Pfizer)	Solution (1 mg/mL of tofacitinib free base)	25 September 2020 (Prescription)	JAK1/2/3 and Tyk2	Rheumatoid arthritis, psoriatic arthritis, ulcerative colitis, and juvenile idiopathic arthritis
	Tablet (5 mg and 10 mg of tofacitinib free base)	6 November 2012 (5 mg) 30 May 2018 (10 mg) (All are prescription products)		
	Extended-release tablet (11 mg and 22 mg of tofacitinib free base)	23 February 2016 (11 mg) 12 December 2019 (22 mg) (All are prescription products)		
Cabozantinib S-malate (Cometriq and Cabometyx, Exelixis)	Capsule (20 mg and 80 mg of cabozantinib free base)	29 November 2012 (Prescription)	RET	MTC, RCC, and HCC
	Tablet (20 mg, 40 mg and 80 mg of cabozantinib free base)	25 April 2016 (Prescription)		
Ponatinib hydrochloride (Iclusig, Ariad Pharmaceuticals)	Tablet (10 mg, 15 mg, 30 mg, and 45 mg of ponatinib free base)	18 December 2020 (10 mg) 14 December 2012 (15 mg and 45 mg) 23 April 2015 (30 mg) (All are prescription products)	BCR-Abl	CML, Ph^+^-ALL, T315I-positive CML or Ph^+^-ALL
Trametinib dimethyl sulfoxide (Mekinist, Novartis Pharmaceuticals)	Tablet (0.5 mg, 1 mg and 2 mg)	29 May 2013 (Prescription) (The 1 mg tablet has been discontinued)	MEK1/2	Metastatic melanoma, NSCLC, and ATC
Dabrafenib mesylate (Tafinlar, Novartis Pharmaceuticals)	Capsule (50 mg and 75 mg of dabrafenib free base)	29 May 2013 (Prescription)	B-Raf	Metastatic melanoma, NSCLC, and ATC
Afatinib dimaleate (Gilotrif, Boehringer Ingelheim)	Tablet (20 mg, 30 mg, and 40 mg of afatinib free base)	12 July 2013 (Prescription)	EGFR/HER2/HER4	NSCLC
Ibrutinib (Imbruvica, Pharmacyclics)	Capsule (70 mg and 140 mg)	13 November 2013 (140 mg) 20 December 2017 (70 mg)	BTK	MCL, CLL, SLL, and MZL
	Tablet (140 mg, 280 mg, 420 mg, and 560 mg)	16 February 2018		
Ceritinib (Zykadia, Novartis Pharmaceuticals)	Tablet (150 mg)	18 March 2019 (Prescription)	ALK	NSCLC
	Capsule (150 mg)	29 April 2014 (Discontinued)		
Idelalisib (Zydelig, Gilead Sciences)	Tablet (100 mg and 150 mg)	23 July 2014 (Prescription)	PI3K_δ_	CLL, FL, and SLL
Nintedanib esylate (Ofev, Boehringer Ingelheim Pharmaceuticals)	Capsule (100 mg and 150 mg of nintedanib free base)	15 October 2014 (Prescription)	PDGFR/FGFR/VEGFR	IPF, ILDs, and SSc-ILD
Palbociclib (Ibrance, Pfizer)	Capsule (75 mg, 100 mg, and 125 mg)	3 February 2015 (Prescription)	CDK4/6	Breast cancer
	Tablet (75 mg, 100 mg, and 125 mg)	1 November 2019 (Prescription)		
Lenvatinib mesylate (Lenvima, Eisai)	Capsule (4 mg and 10 mg of lenvatinib free base)	13 February 2015 (Prescription)	VEGFR/RET	Thyroid cancer, RCC, HCC, and endometrial carcinoma
Cobimetinib fumarate (Cotellic, Genentech)	Tablet (20 mg of cobimetinib free base)	10 November 2015 (Prescription)	MEK1/2	Melanoma
Osimertinib mesylate (Tagrisso, AstraZeneca Pharmaceuticals)	Tablet (40 mg and 80 mg of osimertinib free base)	13 November 2015 (Prescription)	EGFR	NSCLC
Alectinib hydrochloride (Alecensa, Hoffmann-La Roche)	Capsule (150 mg of alectinib free base)	11 December 2015 (Prescription)	ALK/RET	NSCLC
Ribociclib succinate (Kisqali, Novartis Pharmaceuticals)	Tablet (200 mg of ribociclib free base)	13 March 2017 (Prescription)	CDK4/6	Breast cancer
Brigatinib (Alunbrig, Ariad Pharmaceuticals)	Tablet (30 mg, 90 mg, and 180 mg)	28 April 2017 (30 mg, and 90 mg) 2 October 2017 (180 mg) (All are prescription products)	ALK	NSCLC
Midostaurin (Rydapt, Novartis Pharmaceuticals)	Capsule (25 mg)	28 April 2017 (Prescription)	Flt3	AML, MCL, and systemic mastocytosis
Neratinib maleate (Nerlynx, Puma Biotechnology)	Tablet (40 mg of neratinib free base)	17 July 2017 (Prescription)	EGFR/HER2	Breast cancer
Copanlisib dihydrochloride (Aliqopa, Bayer Healthcare Pharmaceuticals)	Powder (60 mg/vial)	14 September 2017 (Prescription)	PI3K-α/β/δ	FL and Non-Hodgkin Lymphoma
Abemaciclib (Verzenio, Eli Lilly)	Tablet (50 mg, 100 mg, 150 mg, and 200 mg)	28 September 2017 (Prescription)	CDK4/6	Breast cancer
Acalabrutinib (Calquence, Astrazeneca)	Capsule (100 mg)	31 October 2017 (Prescription)	BTK	MCL, CLL, SLL, and urothelial carcinoma
Netarsudil mesylate (Rhopressa, Aerie Pharmaceuticals)	Solution/Drops (0.02% of netarsudil free base)	18 December 2017 (Prescription)	ROCK1/2	Open-angle glaucoma or ocular hypertension
Baricitinib (Olumiant, Eli Lilly)	Tablet (1 mg and 2 mg)	8 October 2019 (1 mg) 31 May 2018 (2 mg) (All are prescription products)	JAK1/2/3 andTyk	Rheumatoid arthritis
Binimetinib (Mektovi, Array Biopharma)	Tablet (15 mg)	27 June 2018 (Prescription)	MEK1/2	Melanoma with a BRAF V600 mutation
Dacomitinib (Vizimpro, Pfizer)	Tablet (15 mg, 30 mg, and 45 mg)	27 September 2018 (Prescription)	EGFR/HER1	NSCLC
Encorafenib (Braftovi, Array Biopharma)	Capsule (50 mg, and 75 mg)	27 June 2018 (Prescription) (50 mg capsules have been discontinued)	B-Raf	Melanoma
Fostamatinib disodium (Tubeless, Rigel Pharmaceuticals)	Tablet (100 mg, and 150 mg of fostamatinib free base)	17 April 2018 (Prescription)	Syk	ITP
Duvelisib (Copiktra, Secura Bio)	Capsule (15 mg, and 25 mg)	24 September 2018 (Prescription)	PI3K-δ/PI3K-γ	CLL, SLL, FL, and hematological malignancies
Gilteritinib fumarate (Xospata, Astellas Pharma)	Tablet (40 mg of gilteritinib free base)	28 November 2018 (Prescription)	Flt3	AML
Larotrectinib sulfate (Vitrakvi, Bayer Healthcare Pharmaceuticals)	Capsule (25 mg, and 100 mg of larotrectinib free base)	26 November 2018 (Prescription)	TRK	Solid tumors
	Solution (20 mg of larotrectinib free base per ml)			
Lorlatinib (Lorbrena, Pfizer)	Tablet (25 mg, and 100 mg)	2 November 2018 (Prescription)	ALK	NSCLC
Entrectinib (Rozlytrek, Genentech)	Capsule (100 mg and 200 mg)	15 August 2019 (Prescription)	TRK-A, TRK-B, and TRK-C	NSCLC and solid tumors
Upadacitinib (Rinvoq, Abbvie)	Extended-release tablet (15 mg)	16 August 2019 (Prescription)	JAK	Rheumatoid arthritis
Alpelisib (Piqray, Novartis Pharmaceuticals)	Tablet (50 mg, 100 mg, and 200 mg)	24 May 2019 (Prescription)	PI3K	Breast cancer
Erdafitinib (Balversa, Janssen Biotech)	Tablet (3 mg, 4 mg, and 5 mg)	12 April 2019 (Prescription)	FGFR1/2/3/4	Metastatic urothelial carcinoma (mUC)
Pexidartinib hydrochloride (Turalio, Daiichi Sankyo)	Capsule (200 mg of pexidartinib free base)	2 August 2019 (Prescription)	CSF1R/KIT/Flt3	TGCT
Fedratinib hydrochloride (Inrebic, Impact Biomedicines)	Capsule (100 mg of fedratinib free base)	16 August 2019 (Prescription)	JAK2	Myelofibrosis
Zanubrutinib (Brukinsa, Beigene)	Capsule (80 mg)	14 November 2019 (Prescription)	BTK	MCL, CLL, WM, and SLL
Avapritinib (Ayvakit, Blueprint Medicines)	Tablet (100 mg, 200 mg, and 300 mg)	9 January 2020 (Prescription)	PDGFRA/KIT	GIST
Selumetinib sulfate (Koselugo, Astrazeneca Pharmaceuticals)	Capsule (10 mg and 25 mg of selumetinib free base)	10 April 2020 (Prescription)	MAPK/MEK 1,2	Neurofibromatosis type 1 (NF1)
Pemigatinib (Pemazyre, Incyte)	Tablet (4.5 mg, 9 mg, and 135 mg)	17 April 2020 (Prescription)	FGFR1- 3	Cholangiocarcinoma
Tucatinib (Tukysa, Seagen)	Tablet (50 mg and 150 mg)	17 April 2020 (Prescription)	HER2	Breast cancer
Capmatinib hydrochloride (Tabrecta, Novartis Pharmaceutical)	Tablet (150 mg and 200 mg of capmatinib free base)	6 May 2020 (Prescription)	MET	NSCLC
Selpercatinib (Retevmo, Loxo Oncology)	Capsule (40 mg and 80 mg)	8 May 2020 (Prescription)	RET/VEGFR	NSCLC, and MTC
Ripretinib (Qinlock, Deciphera Pharmaceuticals)	Tablet (50 mg)	15 May 2020 (Prescription)	PDGFRA/KIT	GIST
Pralsetinib (Gavreto, Blueprint Medicines)	Capsule (100 mg)	4 September 2020 (Prescription)	RET	NSCLC, and MTC
Trilaciclib dihydrochloride (Cosela; G1 Therapeutics Inc.,)	Powder for IV injection (300 mg of Trilaciclib free base per vial)	12 February 2021 (Prescription)	CDK4	ES-SCLC
Tepotinib hydrochloride monohydrate (Tepmetko; EMD Serono Inc.,)	Tablet (225 mg of Tepotinib free base)	3 February 2021 (Prescription)	MET	NSCLC
Umbralisib tosylate (Ukoniq; TG Therapeutics)	Tablet (200 mg of Umbralisib free base)	5 February 2021 (Prescription)	PI3K_δ_ and CK1_ε_	MZL, and FL
Tivozanib hydrochloride monohydrate (Fotivda; Aveo Pharmaceuticals)	Capsule (0.89 mg and 1.34 mg of tivozanib free base)	10 March 2021 (Prescription)	VEGFR/PDGFR	RCC
Infigratinib (Truseltiq; QED Therapeutics)	Capsule (25 and 100 mg)	28 May 2021 (Prescription)	FGFR	Cholangiocarcinoma

^#^ Some drugs are multikinase inhibitors.

**Table 3:** Patent number, applicant/assignee, expiry date, and legal status of the cited patents.

S. No.	Drug’s Name	Patent Number	Applicant/Assignee	Expiry Date	Legal Status	Expected Date of Generic Availability in the USA *
1	Imatinib	US5521184A	Ciba Geigy	4 July 2015	Expired	Generic is available
		USRE43932E	Novartis	16 July 2019	Expired	
2	Gefitinib	US5457105A	Zeneca	19 January 2013	Expired	July 2022 due to the Orphan Drug Exclusivity
		US5770599A	Zeneca	5 May 2017	Expired	
3	Erlotinib	USRE41065E	OSI Pharmaceuticals	8 May 2019	Expired	Generic is available
		US6900221B1	OSI Pharmaceuticals	9 May 2021	Litigation	
4	Sorafenib	US7235576B1	Bayer Pharmaceuticals	12 January 2020	Expired	Generic is available
		US8877933B2	Bayer IP	24 December 2027	Patented	
5	Sunitinib	US7125905B2	Sugen Incorporation	15 August 2021	Patented	August 2021
		US6573293B2	Sugen Incorporation	15 August 2021	Patented	
6	Dasatinib	US6596746B1	Bristol-Myers Squibb	28 December 2020	Expired	Generic is available
		US7491725B2	Bristol-Myers Squibb	28 September 2026	Patented	
7	Lapatinib	US8513262B2	Glaxo Group	8 January 2019	Expired	Generic is available
		US7157466B2	Smithkline Beecham	19 November 2021	Patented	
8	Temsirolimus	USRE44768E	Wyeth	15 August 2019	Expired	Generic is available
9	Everolimus	US5665772A	Sandoz	9 March 2020	Expired	Generic is available
10	Nilotinib	US7169791B2	Novartis	4 January 2024	Patented	February 2029
		US8163904B2	Novartis	23 February 2029	Patented	
		US8415363B2	Novartis	18 January 2027	Patented	
11	Pazopanib	US7105530B2	Smithkline Beecham	19 October 2023	Patented	October 2023
		US8114885B2	Glaxosmithkline	19 December 2021	Patented	
12	Vandetanib	USRE42353E	Astrazeneca	27 June 2022	Patented	June 2022
13	Vemurafenib	US8143271B2	Plexxikon Incorporation	21 June 2026	Patented	June 2026
14	Crizotinib	US7858643B2	Agouron Pharmaceuticals	8 October 2029	Patented	October 2029
		US8217057B2	Pfizer	6 November 2029	Patented	
15	Ruxolitinib	US7598257B2	Incyte Corporation	24 December 2027	Patented	June 2028
		US8722693B2	Incyte Corporation	12 June 2028	Patented	
16	Axitinib	US6534524B1	Agouron Pharmaceuticals	29 April 2025	Patented	April 2025
		US8791140B2	Pfizer	14 December 2030	Patented	
17	Bosutinib	USRE42376E	Wyeth	13 April 2024	Patented	April 2024
		US7767678B2	Wyeth	23 November 2026	Patented	
18	Regorafenib	US8637553B2	Bayer Healthcare	16 February 2031	Patented	July 2032
		US9957232B2	Bayer Healthcare	9 July 2032	Patented	
19	Tofacitinib	USRE41783E	Pfizer	8 December 2025	Patented	December 2025
		US6965027B2	Pfizer	25 March 2023	Patented	
20	Cabozantinib	US7579473B2	Exelixis	14 August 2026	Patented	August 2026
		US8877776B2	Exelixis	8 October 2030	Patented	
21	Ponatinib	US8114874B2	Ariad Pharmaceuticals	24 January 2027	Patented	January 2027
		US9493470B2	Ariad Pharmaceuticals	12 December 2033	Patented	
22	Trametinib	US7378423B2	Japan Tobacco	29 May 2027	Patented	May 2027
23	Dabrafenib	US7994185B2	Glaxo Smith Kline	20 January 2030	Patented	January 2030
24	Afatinib	USRE43431E	Boehringer Ingelheim	13 January 2026	Patented	January 2026
		US8426586B2	Boehringer Ingelheim	10 October 2029	Patented	
25	Ibrutinib	US8735403B2	Pharmacyclics	28 December 2026	Patented	December 2026
		US9296753B2	Pharmacyclics	30 October 2033	Patented	
26	Ceritinib	US8039479B2	IRM	29 June 2030	Patented	June 2030
		US9309229B2	Novartis	18 January 2032	Patented	
27	Idelalisib	USRE44638E	ICOS Corporation	5 August 2025	Patented	August 2025
		US9469643B2	Gilead	2 September 2033	Patented	
28	Nintedanib	US6762180B1	Boehringer Ingelheim	1 October 2025	Patented	October 2025
		US7119093B2	Boehringer Ingelheim	21 February 2024	Patented	
29	Palbociclib	USRE47739E	Warner Lambert	5 March 2027	Patented	5 March 2027
		US10723730B2	Pfizer	8 February 2034	Patented	
30	Lenvatinib	US7253286B2	Eisai	19 October 2021	Patented	October 2021
		US7612208B2	Eisai	19 September 2026	Patented	
31	Cobimetinib	US7803839B2	Exelixis	10 November 2029	Patented	November 2029
		US10590102B2	Exelixis	30 June 2036	Patented	
32	Osimertinib	US8946235B2	Astrazeneca	8 August 2032	Patented	August 2032
33	Alectinib	US9126931B2	Chugai Pharmaceutical	29 May 2031	Patented	May 2031
34	Ribociclib	US8415355B2	Astex Therapeutics	19 February 2031	Patented	19 February 2031
		US9193732B2	Astex Therapeutics	9 November 2031	Patented	
35	Brigatinib	US9012462B2	Ariad Pharmaceuticals	31 July 2030	Patented	July 2030
		US10385078B2	Ariad Pharmaceuticals	10 November 2035	Patented	
36	Midostaurin	US5093330A	Ciba Geigy	21 July 2009	Expired	October 2024
		US7973031B2	Novartis	17 October 2024	Patented	
37	Neratinib	US7399865B2	Wyeth	29 December 2025	Patented	December 2025
38	Copanlisib	USRE46856E	Bayer	22 October 2029	Patented	March 2032
		US10383876B2	Bayer	29 March 2032	Patented	
39	Abemaciclib	US7855211B2	Eli Lilly	15 December 2029	Patented	December 2029
40	Acalabrutinib	US9290504B2	Merck	11 July 2032	Patented	July 2032
		US9796721B2	Acerta Pharma	1 July 2036	Patented	
41	Netarsudil	US8394826B2	Aerie Pharmaceuticals	10 November 2030	Patented	March 2034
		US9415043B2	Aerie Pharmaceuticals	14 March 2034	Patented	
42	Baricitinib	US8158616B2	Incyte Corporation	8 June 2030	Patented	June 2030
43	Binimetinib	US7777050B2	Array Biopharma	13 March 2023	Patented	June 2025 based on ODE
		US9562016B2	Array Biopharma	18 October 2033	Patented	
44	Dacomitinib	US7772243B2	Warner Lambert	26 August 2028	Patented	August 2028
45	Encorafenib	US8501758B2	IRM	4 March 2031	Patented	March 2031
46	Fostamatinib	US7449458B2	Rigel Pharmaceuticals	4 September 2026	Patented	4 September 2026
		US8163902B2	Rigel Pharmaceuticals	17 June 2026	Patented	
47	Duvelisib	US8193182B2	Intellikine	13 February 2030	Patented	February 2030
		USRE46621E	Infinity Pharmaceuticals	17 May 2032	Patented	
48	Gilteritinib	US8969336B2	Astellas Pharma	27 January 2031	Patented	January 2031
49	Larotrectinib	US9127013B2	Array Biopharma	21 October 2029	Patented	October 2029
		US10172861B2	Array Biopharma	16 November 2035	Patented	
50	Lorlatinib	US8680111B2	Pfizer	5 March 2033	Patented	March 2033
		US10420749B2	Pfizer	27 July 2036	Patented	
51	Entrectinib	US8299057B2	Nerviano Medical Sciences	1 March 2029	Patented	March 2029
		US10738037B2	Nerviano Medical Sciences	18 May 2037	Patented	
52	Upadacitinib	USRE47221E	Abbvie	1 December 2030	Patented	December 2030
		US9951080B2	Abbvie	17 October 2036	Patented	
53	Alpelisib	US8227462B2	Novartis	28 September 2030	Patented	September 2030
54	Erdafitinib	US8895601B2	Astex Therapeutics	22 May 2031	Patented	May 2031
55	Pexidartinib	US9169250B2	Plexxikon	21 November 2027	Patented	November 2027
		US9802932B2	Plexxikon	5 May 2036	Patented	
56	Fedratinib	US7528143B2	Targegen	16 December 2026	Patented	December 2026
57	Zanubrutinib	US9447106B2	Beigene	22 April 2034	Patented	April 2034
58	Avapritinib	US9944651B2	Blueprint Medicines Corporation	15 October 2034	Patented	October 2034
59	Selumetinib	US7425637B2	Array Biopharma	11 April 2024	Patented	April 2027 based on ODE
		US9156795B2	Array Biopharma	12 December 2026	Patented	
60	Pemigatinib	US9611267B2	Incyte Corporation	30 January 2035	Patented	January 2035
61	Tucatinib	US8648087B2	Array Biopharma	12 April 2031	Patented	April 2031
62	Capmatinib	US7767675B2	Incyte Corporation	19 November 2027	Patented	June 2031
		US8420645B2	Incyte Corporation	5 June 2031	Patented	
63	Selpercatinib	US10112942B2	Array Biopharma	10 October 2037	Patented	October 2037
		US10584124B2	Array Biopharma	10 October 2038	Patented	
64	Ripretinib	US8461179B1	Deciphera Pharmaceuticals	7 June 2032	Patented	June 2032
65	Pralsetinib	US10030005B2	Blueprint Medicines Corporation	1 November 2036	Patented	November 2036
66	Trilaciclib	US8598186B2	G1 Therapeutics	25 October 2031	Patented	October 2031
67	Tepotinib	US8580781B2	Merck	19 March 2030	Patented	March 2030
		US8329692B2	Merck	30 October 2029	Patented	
68	Umbralisib	US10570142B2	Rhizen Pharmaceuticals	2 July 2033	Patented	July 2033
		US10414773B2	Rhizen Pharmaceuticals	26 May 2035	Patented	
69	Tivozanib	US6821987B2	Kirin Beer Kabushiki Kaisha	26 April 2022	Patented	10 March 2026, based on NCE (Patent term extension is possible)
		US7211587B2	Kirin Beer Kabushiki Kaisha	26 April 2022	Patented	
		US7166722B2	Kirin Beer Kabushiki Kaisha	21 October 2023	Patented	
70	Infigratinib	US8552002B2	Novartis	13 December 2025	Patented	25 May 2026, based on NCE (Patent term extension is possible)
		US9067896B2	Novartis	24 February 2031	Patented	

* Based on the patent expiry date.

## Data Availability

Data sharing not applicable.

## Abbreviations

ALK: Anaplastic lymphoma kinase; ALL: Acute lymphoblastic leukemia; AML: Acute myelogenous leukemia; API: Active pharmaceutical ingredient; ARCC: Advanced renal cell carcinoma; ATC: Anaplastic thyroid cancer; ATP: Adenosine triphosphate; BCR-Abl: Breakpoint cluster region/-abl oncogene; BRAF/B-raf: Murine sarcoma viral oncogene homolog; BTK: Bruton’s tyrosine kinase; CDK: Cyclin-dependent protein kinase; CLL: Chronic lymphocytic leukemia; CML: Chronic myelonoid leukemia; CSF1R: Colony stimulating factor 1 receptor; DTC: Differentiated thyroid cancer; EGFR: Epidermal growth factor receptor; FKBP12/mTOR: FK Binding Protein-12/mammalian target of rapamycin; FL: Follicular lymphoma; Flt3: fms-like tyrosine kinase 3; GISTs: Gastrointestinal stromal tumors; GTP: Guanosine triphosphate; HCC: Hepatocellular carcinoma; HER-1/HER-2: Human epidermal growth factor receptor ½; HGFR: Hepatocyte growth factor receptor; ILDs: Interstitial lung disease; IPF: Idiopathic pulmonary fibrosis; ITP: Immune thrombocytopenic purpura; JAK: Janus kinase; MAPK/MEK1/2: Mitogen-activated protein kinase kinase; MAT: Monoamine transporter; MCL: Mantle cell lymphoma; MTC: Medullary thyroid cancer; mUC: Metastatic urothelial carcinoma); MZL: Marginal zone lymphoma; NF1: Neurofibromatosis type 1; NSCLC: Non-small cell lung cancer; PDGFR: Platelet-derived growth factor receptor; Ph^+^-ALL: Philadelphia chromosome-Positive Acute lymphoblastic leukemia; Ph^+^-CML: Philadelphia chromosome-positive chronic myeloid leukemia; PI3K: Phosphatidylinositol 3-kinase; PKIs: Protein kinase inhibitors; PKs: Protein kinases; pNET: Primitive neuroectodermal tumor; RCC: Renal cell carcinoma; SLL: Small lymphocytic lymphoma; SSc: Systemic sclerosis; STS: Soft-tissue sarcomas; TKI: Tyrosine Kinase inhibitors; Tyk2: Tyrosine kinase; USFDA: United States Food and Drug Administration; VEGFR: Vascular endothelial growth factor receptor.
